# Identification and experimental validation of prognostic genes associated with dietary restriction-related signatures in bladder cancer

**DOI:** 10.3389/fimmu.2026.1891824

**Published:** 2026-09-11

**Authors:** Xian Wu, Tianshui Sun, Zhuonan Liu

**Affiliations:** 1 Department of Urology, First Hospital of China Medical University, Heping District, Shenyang, Liaoning, China; 2 Department of Obstetrics and Gynecology, Shengjing Hospital of China Medical University, Shenyang, Liaoning, China

**Keywords:** bladder cancer, DRRGs, MR analysis, nomogram, risk model, ScRNA-seq analysis

## Abstract

**Background:**

Bladder cancer (BLCA) is a common urinary tract malignancy with significant global burden, yet the prognostic relevance of dietary restriction-related genes (DRRGs) in BLCA remains unclear.

**Methods:**

Prognostic DRRGs in BLCA were identified based on publicly available databases by differential expression analysis, univariate Cox regression analysis, Mendelian randomization (MR) analysis and LASSO regression analysis. A risk model was built and evaluated, after which a nomogram was established and assessed by using the risk score and clinical characteristics. Following this, analysis of functional enrichment, immune infiltration, genetic mutation and drug sensitivity were performed based on the risk model. Importantly, cell annotation and identification of potential key cells were conducted by single-cell RNA sequencing (scRNA-seq) analysis. The expression levels of target DRRGs were examined by qRT-PCR and western blot methods. Cell transfection, *in vivo* and *in vitro* assays were performed to analyze the impacts of target DRRGs on BLCA cells’ biological behaviors.

**Results:**

Three candidate prognostic DRRGs (RBP7, SREBF1 and ZFP64) were screened. The risk model classified BLCA patients two risk groups, and high-risk patients showed worse survival. The AUC values of this model in the training and validation cohorts were generally within the range of 0.60–0.66, suggesting some but limited discriminatory ability. The risk score, age, T stage, and N stage were identified as independent prognostic factors, and the nomogram constructed from these factors showed moderate predictive performance. The two risk groups differed in signaling pathways, immune cell infiltration, immune checkpoints, mutated genes, immunotherapy-related predictive indicators, and drug sensitivity. scRNA-seq analysis annotated eight cell types, among which T cells showed differential expression of the candidate DRRGs and were identified as a potential key cell type associated with prognostic DRRG expression. Experiments showed that SREBF1 and ZFP64 were over-expressed at mRNA and protein levels in BLCA tissues. Knockdown of SREBF1 or ZFP64 inhibited multiple malignant phenotypes of BLCA cells.

**Conclusions:**

This study screened RBP7, SREBF1, and ZFP64 from a previously computationally inferred DR-related gene category as candidate prognostic genes in BLCA and established a risk model with moderate predictive performance, providing clues for further investigation of this malignancy.

## Introduction

As one of the most common malignancies of the urinary system, bladder cancer (BLCA) causes considerable morbidity and mortality (1, 2). Urothelial carcinoma is the predominant pathological subtype of BLCA, which is characterized by high recurrence and progression rates (2, 3). Muscle–invasive bladder carcinoma (MIBC), associated with higher metastatic probability and worse prognosis, is observed in approximately 25% of newly diagnosed BLCA patients (4). Over the past decade, despite considerable advances in chemotherapy, immunotherapy, and targeted therapy for BLCA, issues such as low objective response rates and drug resistance remain significant concerns. In addition, a lack of individualized treatment is another reason for unsatisfactory outcomes (5). Thus, there is an urgent need to identify more biomarkers for this neoplasm and to characterize their molecular functions and mechanisms, thereby guiding improved therapeutic strategies.

Dietary restriction (DR), also known as calorie restriction (CR), is a general concept referring to restriction of energy intake without causing malnutrition (6). As early as 2010, DR was identified as an effective non-pharmacological method for cancer prevention in rodents and monkeys, and it was speculated to have a similar anticancer function in humans based on its inhibition on production of growth factors, inflammatory cytokines, anabolic hormones, oxidative stress, and free radical-induced DNA damage (7). Subsequently, the associations of DR with tumor prevention and treatment have been further determined, and it was reported that dietary patterns could predict BLCA risks (8, 9). Recently, Song et al. evaluated DR-related features at a pan-cancer level based on previously curated dietary restriction-related transcriptional programs using GSVA/ssGSEA along with other methods and constructed a gene set containing 276 dietary restriction-related genes (DRRGs) (10). That study shows that DRRG signature activity is associated with tumor immune status and immunotherapy-response predictive indicators and suggests that some DRRGs may have prognostic assessment value (10). However, not every member of this gene set has been experimentally validated as a mechanistic effector gene under dietary restriction intervention. Nevertheless, these DRRGs provide a valuable candidate scope for exploring tumor prognostic molecules. In BLCA, however, the prognostic significance of these DR-related transcriptional feature genes and their potential links with the tumor microenvironment and tumor-cell biological behavior remain to be further investigated.

In recent years, developments in Mendelian randomization (MR) analysis and sequencing technologies provide new approaches for screening tumor-related candidate molecules. MR analysis uses genetic variants as instrumental variables and can, to some extent, reduce the influence of confounding factors and reverse causality; it has been applied to explore BLCA-related molecular features and potential risk factors (11–14). Meanwhile, bulk RNA-seq can characterize tumor-related expression changes at the overall transcriptomic level, whereas single-cell RNA sequencing (scRNA-seq) can further resolve tumor heterogeneity and microenvironmental features at a cell-type-specific level (15–17). Therefore, integrating transcriptomic analysis, genetic evidence, single-cell analysis, and experimental validation may help to more systematically identify candidate molecules associated with BLCA prognosis and preliminarily explore their potential biological significance.

Based on this background, this study uses previously computationally inferred DR-related transcriptional features as the source of candidate genes and explores candidate molecules related to BLCA prognostic stratification, the tumor immune microenvironment, and tumor-cell biological behavior. We hypothesize that some DR-related transcriptional feature genes may participate in BLCA prognosis-related phenotypes and may have further research value. To this end, this study integrates public transcriptomic data, genetic analysis, prognostic model construction, scRNA-seq analysis, and preliminary experimental validation to screen and evaluate three candidate genes, RBP7, SREBF1, and ZFP64. Overall, this study aims to provide candidate molecular clues for BLCA prognostic assessment and subsequent mechanistic research, and to offer a reference for understanding the potential link between DR-related transcriptional features and BLCA biological characteristics.

## Materials and methods

### Data collection

The transcriptome, clinical information, and survival data for bladder cancer (BLCA) were sourced from UCSC Xena database (accessed on October 23, 2024) (https://xenabrowser.net/datapages/). For the TCGA-BLCA cohort, tumor samples with missing overall survival (OS) status or overall survival time were excluded before survival-related analysis and model construction; finally, 404 BLCA tumor tissue samples with complete survival data were retained as the BLCA group. Meanwhile, 18 adjacent normal tissue samples were included as the control group. For other clinical variables, including age, sex, T stage, and N stage, missing values were retained as NA. Meanwhile, GSE13507 (platform: GPL6102) and GSE222315 (platform: GPL24676) were retrieved from the GEO database (https://www.ncbi.nlm.nih.gov/geo/). GSE13507 comprised BLCA tumor tissue samples from 165 primary BLCA patients with complete survival information. The single cell dataset GSE222315 consisted of 9 BLCA tumor tissue samples and 4 control paraneoplastic tissue samples. Moreover, 276 dietary restriction-related genes (DRRGs) were downloaded by reviewing the literature (10) (**Supplementary Table 1**). Furthermore, the GWAS data of BLCA (ieu-b-4874) were retrieved utilizing the IEU OpenGWAS database, including 373,295 European samples, 1,279 cases, and 372,016 control samples, 9,904,926 single nucleotide polymorphisms (SNPs). Finally, the expression quantitative trait locus (eQTLs) of exposure factors were also obtained from IEU OpenGWAS database.

### Acquisition of differentially expressed genes (DEGs)

In this study, the “DESeq2” package (v 1.38.0) (18) was used to obtain the DEGs between BLCA and control groups (BLCA vs Control) in TCGA-BLCA. Screening was based on |log2FoldChange(FC)| > 0.5, p< 0.05 (19). Meanwhile, in order to understand the distribution of DEGs, the “ggplot2” package (v 3.4.4) (20) was used to draw volcano plot to visualize the DEGs, and the top 5 up- and down-regulated genes according to log2FC from high to low were marked in the volcano map. At the same time, heatmap was plotted via “ComplexHeatmap” package (v 2.14.0) (21) to show up- and down-regulated top10 genes in DEGs. Following this, the “VennDiagram” (v 1.7.3) (22) was employed to identify the candidate genes by overlapping the DRRGs and DEGs.

### Enrichment analysis of candidate genes

Gene Ontology (GO) and Kyoto Encyclopedia of Genes and Genomes (KEGG) analyses were conducted on candidate genes using “clusterProfiler” package (v 4.7.1.003) (23) and *p* < 0.05 was considered statistically significant. Pathways with top5 p-values of significance for each section of GO analysis were selected for visualization. For KEGG enrichment analysis, top15 pathways sorted by p-value (in ascending order) were selected for visualization. A protein-protein interaction (PPI) network (interaction scores > 0.4) was created by STRING online database (https://string-db.org), drawn via “Cytoscape” (v 3.9.1) (24).

### Identification of prognostic-related genes

In TCGA-BLCA, based on the candidate genes, proportional hazards (PH) assumption tests (*p* > 0.05), univariate Cox regression analysis [hazard ratio (HR) ≠ 1 and p< 0.05), multivariate PH assumption tests (*p* > 0.05) and multivariate Cox regression analysis were performed out to identify prognostic-related genes by “survminer” package (v 0.4.9) (25). The results were displayed in forest plot by “forestplot” package (v 3.1.1) (https://CRAN.R-project.org/package=forestplot).

### MR analysis

To explore the potential causal association between prognosis-related genes and BLCA, we conducted MR analysis with prognostic-related genes as the exposure factors and BLCA as the outcome variable. BLCA outcome data were obtained from the IEU OpenGWAS database (ieu-b-4874), comprising 373,295 European individuals, including 1,279 BLCA cases and 372,016 controls. The eQTL data for exposures were obtained from the eQTLGen Consortium (n = 31,684, European population), a dataset primarily based on whole-blood samples to assess genetic variants associated with gene expression.

In the main MR analysis, the “TwoSampleMR” package (v 0.5.7) (26) first was carried out to read the exposure factors and screen the instrumental variables (IVs), to find the IVs with significant correlation with the exposure factors. The screening process included: p< 5×10^-7^ (27), looking for SNPs significantly associated with exposure factors; clump = TRUE, r^2^ = 0.001, and kb = 50, removing the SNP of linkage imbalance (LD); F statistic > 10, removing weak IVs; exposure factors with fewer than three SNPs were excluded. Subsequently, the outcome was read by “TwoSampleMR” package (v 0.5.7) and screened for IVs in relation to exposure factors and not associated with outcome by combining them with IVs from the exposure factors.

To clarify the source of instrumental variables and their cis/trans attributes, we further traced the instrumental variables actually included in the main MR analysis. Specifically, the chromosomal position of each gene-specific instrumental SNP was extracted, and the transcription start site (TSS) of the corresponding gene was obtained based on GRCh37 genome annotation. The distance between each SNP and the corresponding gene TSS was then calculated. SNPs located within the TSS ±1 Mb window were defined as cis-eQTLs, whereas SNPs outside this window were defined as trans-eQTLs.

Then, the harmonize_data function in “TwoSampleMR” package (v 0.5.7) was applied to harmonize the effect alleles and effect sizes. MR analysis was conducted via a combination of 5 algorithms, including MR Egger (28), Weighted median (29), Inverse variance weighted (IVW) (30), Simple mode (31) and Weighted mode (32), in which IVW was the main analysis method. In the list of MR analysis results, we needed to follow: the *p* < 0.05 of IVW indicated that there was a potential causal association between the exposure factor and the outcome, the rest of the p-values were not mandatory. Then, the odds ratio (OR) >1 was considered a risk factor, and OR< 1 was considered a protective factor. Results were presented through scatter plots, forest plots, and funnel plots. In scatter plots, a positive slope of the line was indicative of a risk factor and a negative slope of the line was indicative of a protective factor. If there was an intercept, this was an indication that there were confounding factors. In forest plots, gene with effect sizes to the left of the dashed line was considered protective factor, while on the opposite side was considered a risk factor. In funnel plots, if the distribution of SNPs on both sides of the IVW line was essentially homogeneous and relatively symmetrical from left to right, the MR analysis aligned with Mendel’s second law.

For assessing the dependability of MR analysis results, sensitivity analyses were carried out. Heterogeneity was analyzed by function mr_heterogeneity of the “TwoSampleMR” package (v 0.5.7) (IVW regression method was used to calculate Cochran’s Q-statistic, which indicated that there was no heterogeneity when *p* > 0.05). Horizontal multiple validity test was carried out with “TwoSampleMR” package (v 0.5.7) function mr_pleiotropy_test and “MRPRESSO” package (v 1.0) (33). When *p* > 0.05, it indicated that there was no significant horizontal pleiotropy. Leave-one-out (LOO) test was carried out with “TwoSampleMR” package (v 0.5.7) function mr_leaveoneout to show that the results of MR analyses were reliable. Finally, to detect possible directions of causality between exposure factors and outcomes, MRsteiger filtering analyses were performed based on the IVW approach described above (correct causal direction, and *p* < 0.05). After that, genes with univariate Cox regression analysis results and MR results in the same direction were selected as candidate prognostic genes.

Finally, to verify the robustness of the MR results, we further extracted instrumental variables using a more stringent threshold of *p* < 5 × 10–^8^ while keeping the remaining parameters unchanged, and repeated the instrumental-variable screening, five MR methods, heterogeneity testing, horizontal pleiotropy testing, and F-statistic evaluation.

### Identification of prognostic genes and risk model construction

On the basis of candidate prognostic genes, the LASSO method was carried out with “glmnet” package (v 4.1.4) (34). LASSO regression was used to shrink the regression coefficients of the candidate genes and to construct a weighted prognostic risk score. The optimal lambda value was determined by 10-fold cross-validation, and genes with nonzero coefficients at lambda.min were retained for model construction. To further evaluate the internal stability of the prognostic model and estimate potential optimism, 1,000 bootstrap resamples were performed in the training cohort. In each resampling iteration, the model was refitted, and the apparent C-index in the bootstrap sample and the test C-index in the original dataset were calculated separately. Optimism was defined as the difference between the apparent C-index in the bootstrap sample and the test C-index in the original dataset, and the optimism-corrected C-index was obtained by subtracting the mean optimism from the apparent C-index of the original model. In addition, to assess the agreement between model-predicted survival probabilities and observed survival probabilities, patients were divided into four groups according to risk score quartiles, and 3-, 5-, and 7-year calibration curves were plotted.

Finally, for the finalized prognostic genes, the Benjamini–Hochberg (BH)-adjusted *p* values (adjusted *p* values) from the TCGA-BLCA differential expression analysis were further determined to evaluate the stability of these genes after correction for multiple testing.

A risk model was created using prognostic genes. The risk score was determined by the following formula:


$$\text{risk score}=\sum_{i=1}^{n}\text{coef}\left({\text{gene}}_{\text{i}}\right)\times \exp r\left({\text{gene}}_{\text{i}}\right)$$

where “coef” represented the coefficients of the prognostic genes in LASSO and “expression” indicated their expression levels.

After that, to evaluate the risk model, 404 BLCA patients in TCGA-BLCA were stratified into high risk group (HRG) and low risk group (LRG) using the median of the risk score. Kaplan-Meier (K-M) curves for the HRG and LRG were drawn by “survminer” package (v 0.4.9). The differences of overall survival (OS) between HRG patients and LRG patients were analyzed by log-rank test (*p* < 0.05). In addition, the survivalROC package (v 1.0.3.1) (35) was used to plot receiver operating characteristic (ROC) curves for 3-, 5-, and 7-year survival, and the areas under the curves (AUCs) were calculated. To quantify the uncertainty of the AUC estimates, 95% confidence intervals for the 3-, 5-, and 7-year time-dependent AUCs in the training set were calculated using the 500-time bootstrap percentile method. Specifically, the original samples were resampled with replacement, with each resampled dataset having the same sample size as the original dataset; the AUC at each time point was recalculated in each bootstrap sample, and after 500 repetitions, the 2.5th and 97.5th percentiles of the bootstrap AUC distribution were used as the 95% confidence interval (AUC > 0.6).

Moreover, risk curves showing the distribution of risk scores and scatter diagrams depicting survival states for HRG and LRG patients were generated, and an expression heatmap of the prognostic genes in both risk groups was also created. Notably, the risk model was also validated in the GSE13507 dataset (165 samples with complete survival information), and the confidence intervals of the AUC values were likewise calculated using the bootstrap percentile method.

### Independent prognostic analysis

In BLCA patients with complete survival information from TCGA-BLCA, we included risk score and 4 clinical characteristics (age, gender, T stage, and N stage) for independent prognostic analysis. The PH assumption tests (p > 0.05) and univariate Cox regression (HR ≠ 1, *p* < 0.05) of risk scores and clinical characteristics were carried out with “survminer” package (v 0.4.9). Subsequently, PH assumption tests (p > 0.05) and multivariate Cox regression were carried out to evaluate independent prognostic factors (HR ≠ 1, *p* < 0.05). Based on independent prognostic factors, a nomogram was developed by “rms” (v 6.5.9) (36) to predict 3-, 5-, and 7-year survival probabilities for BLCA patients. To evaluate the discriminative ability of the model, the survivalROC package (v 1.0.3.1) (37) was used to plot ROC curves, and the 95% confidence intervals of the 3-, 5-, and 7-year AUCs were calculated using the 500-time bootstrap percentile method described above; calibration curves were also plotted using the rms package (v 6.5.9). In addition, decision curve analysis (DCA) was performed using ggDCA (v 1.2) to compare the net benefit of the risk score model, clinical staging model, and combined nomogram model across different threshold probabilities. Harrell’s concordance index (C-index) was also calculated to evaluate model discrimination. Brier score, IPCW-corrected Brier score, calibration slope, and calibration intercept were further calculated to quantitatively assess the agreement between model-predicted survival probabilities and observed survival outcomes.

### Stratified analysis of the clinical characteristics

To further determine the relationship between risk scores and clinical characteristics, including age (> 60 and ≤ 60), gender (female and male), T stage (T1/2, T3 and T4), and N stage (N0, N1 and N2/3) of BLCA, the Wilcoxon test was carried out to explore the differences in risk scores across different clinical characteristic subgroups in TCGA-BLCA (p< 0.05). In addition, heatmaps were plotted by “pheatmap” package (v 1.0.12) (38) to show prognostic gene expression in clinical characteristic subgroups and risk groups (HRG and LRG).

### Gene set enrichment analysis (GSEA)

To understand the biological processes between HRG and LRG, GSEA was performed in TCGA-BLCA. The “DESeq2” package (v 1.38.0) was used to analyze the differences between 2 groups (HRG vs LRG), the log2FC values of the genes were calculated and organized in descending order, and using “clusterProfiler” package (v 4.7.1.003) to carry out GSEA (|NES| > 1, *p* < 0.05). And “h.all.v2023.2.Hs.symbols.gmt” was retrieved from the MSigDB (https://www.gsea-msigdb.org/gsea/msigdb) to serve as background gene set. Subsequently, then the pathways were sorted in descending order of NES, the top 3 pathways were visualized.

### Immunological microenvironmental analysis

In TCGA-BLCA, to examine immune cell infiltration in HRG and LRG, an immune infiltration analysis was conducted. The ssGSEA algorithm was utilized to compute infiltration scores for 28 cell types (39) via “GSVA” (v 1.46.0) (40). These infiltration scores were compared between 2 groups using the Wilcoxon test, identifying immune cells with significant differences (p< 0.05) for further investigation. Subsequently, Wilcoxon test (*p* < 0.05) was used to analyze differences in immune checkpoint (41) expression between 2 risk groups. Spearman correlation analysis was performed using the psych package (v 2.2.9) (42) to examine the relationships between prognostic genes and differentially infiltrated immune cells, as well as between prognostic genes and differentially expressed immune checkpoints. The Benjamini–Hochberg method was used to correct the *p* values from the correlation tests for multiple comparisons; correlations were considered significant when |correlation coefficient (cor)| > 0.3 and the BH-adjusted *p* value was< 0.05.

### Somatic mutation analysis

The “maftools” package (v 2.14.0) (43) was used to analyze somatic mutations in BLCA patients in HRG and LRG of TCGA-BLCA. The top 20 genes were identified based on mutation frequency, waterfall plots were generated to visualize these results. Additionally, waterfall plots were also drawn through the “maftools” package (v 2.14.0) based on prognostic genes.

### Analysis of immunotherapy-related predictive indicators and drug sensitivity prediction

To explore differences in immunotherapy-related predictive pathways between the HRG and LRG, immunotherapy-related predictive pathways were first obtained from the literature (44). Then, based on the two patient groups, the activity of immunotherapy-related predictive pathways was evaluated using the ssGSEA method implemented in the GSVA package (v 1.46.0), and differences in pathway activity between the HRG and LRG were tested using the Wilcoxon test (*p* < 0.05). Meanwhile, ESTIMATE algorithm was utilized for the calculation of stromal scores, immune scores, and ESTIMATE scores for patients in HRG and LRG. Furthermore, targeted immunomodulatory drug enhancement (TIDE, http://tide.dfci.harvard.edu/) was performed for the calculation of TIDE prediction scores for patients in HRG and LRG. Thereafter, the relationship between risk scores and TIDE prediction scores was evaluated using Spearman correlation analysis (|cor| > 0.3 and BH-adjusted *p<* 0.05). And Wilcoxon test was performed to investigate differences in above scores between HRG and LRG (*p* < 0.05). The above results were plotted by the “ggstatsplot” package (v 0.11.0) (45) and “ggpubr” package (v 0.6.0), respectively (46). It should be emphasized that this study did not include an actual immunotherapy cohort; therefore, the related scores cannot be directly equated with clinical treatment responses.

To infer the potential sensitivity of patients in the two risk groups to chemotherapeutic drugs, the “pRRophetic” package (v 0.5) (47) was used to calculate the half-maximal inhibitory concentration (IC50) of 138 therapeutic agents for each BLCA sample in TCGA-BLCA. The differences in IC50 values between 2 groups for the therapeutic agents were analyzed by Wilcoxon test (*p* < 0.05).

### ScRNA-seq analysis

Data from BLCA and control samples in the GSE222315 dataset were filtered for scRNA-seq analysis by “Seurat” (v 5.0.1) (48). Low-expression cells and genes were eliminated by the following filtration criteria, (1) low quality cells with genes less than 200; (2) abnormally highly expressed cells with genes greater than 6000; (3) the sum of the expression of all genes measured per cell greater than 20,000; (4) cells with percentage of mitochondria greater than 10%. The above filtered data were normalized by the LogNormalize to obtain the standardized data. Then, the FindVariableFeatures function was carried out to select the top 2,000 variable genes. Following this, a principal component analysis (PCA) plot was drawn between all the samples to determine whether there were clear boundaries between the samples. At the same time, these 2,000 variable genes were employed for conducting PCA. Then, the JackStrawPlot function was specifically utilized to compare the distribution of *p*-values for each principal components (PCs) (*p* < 0.05).

To assess whether the clustering results depended on a single resolution parameter, we further performed clustering robustness tests at different resolutions. In addition to the original analysis at resolution = 0.5, resolutions of 0.1, 0.3, 0.7, and 1.0 were additionally tested. Cell clustering and UMAP visualization were repeated separately at each resolution.

The ElbowPlot function was employed to visualize the scree plot, and the number of PCs corresponding to where the curve began to level off were selected for uniform manifold approximation and projection (UMAP) cell cluster analysis (resolution =0.5). Later, we annotated the cell clusters based on the marker genes in the literature (49), bubble plots depicting marker gene expression in different cell types were created using the “ggplot2” package (v 3.4.4). The proportion of each cell type in BLCA and control groups was displayed using a bar plot. Following this, these cell types with significant difference between these 2 groups (*p* < 0.05) were selected using the Wilcoxon test and named as differential cell types. Simultaneously, the “ReactomeGSA” package (v 1.12.0) (50) was carried out to perform functional enrichment analysis for differential cell types in GSE222315, and heat mapping was performed based on the top15 scoring differences.

### Identification of the potential key cell type related to prognostic DRRG expression

In GSE222315, UMAP plots were created to show prognostic genes expression in all cell types. Prognostic gene expression was analyzed in all cell types of BLCA and control samples using the Wilcoxon test (*p* < 0.05). Cells with differentially expressed prognostic genes and belonging to differential cell types were selected for key cells.

During this process, because cell populations annotated as epithelial cells in tumor samples may, in certain circumstances, contain both malignant and non-malignant epithelial components, we further used CopyKAT to infer malignant cell identity based on copy-number variation features. Specifically, immune cells from the same single-cell dataset, including T cells, B cells, and NK cells, as well as endothelial cells, were used as relative diploid reference cells to classify the epithelial-cell population as aneuploid or diploid. The numbers and proportions of aneuploid cells among epithelial cells from BLCA and control samples were then calculated separately to assess whether malignant epithelial cells with copy-number abnormality features were present among epithelial cells from tumor samples.

### Reannotation of potential key cell subclusters and assessment of functional states

To further characterize the heterogeneity of potential key cells and their relationship with prognostic DRRG expression, the annotated potential key cells were extracted from the GSE222315 single-cell dataset and were renormalized, dimensionally reduced, clustered, and annotated at the subcluster level. Potential key-cell subclusters were characterized according to classical functional markers, including the pan-T-cell marker CD3D, the CD4 T-cell marker CD4, the CD8 T-cell marker CD8A, the regulatory T-cell-associated marker FOXP3, and immune checkpoint/exhaustion-associated markers such as PDCD1, CTLA4, and HAVCR2. Bubble plots were then generated to show the expression patterns of these markers across different T-cell subclusters and to assess the functional states of the T-cell subclusters. Bubble plots and heatmaps were further generated to show the distribution of prognostic gene expression across different T-cell subclusters.

### Cell communication, heterogeneity and pseudo-time analyzes

The communication network between cells was carried out with the “CellChat” package (v 1.6.1) (51). To gain deeper insights into heterogeneity of the potential key cells, the cells in GSE222315 were regrouped (resolution=0.4) and annotated into different cell subsets. The clustering results were visualized using UMAP. In order to investigate the differentiation trajectory and evolution of the potential key cells during development, the “Monocle” package (v 2.26.0) (52) had been used for pseudo-time analysis.

### Clinical samples

All the clinical samples were obtained from patients who had been diagnosed with bladder urothelial carcinoma and undergone radical cystectomy in the First Hospital of China Medical University. 20 pairs of tumor tissues and their corresponding adjacent normal tissues were used for quantitative real time PCR (qRT-PCR) assay, while 10 pairs were examined by western blot assay. The adjacent normal tissues were more than 3 cm from the tumor lesions. All participants provided written informed consent and the Research Ethics Committee of the First Hospital of China Medical University approved the use of the clinical samples (NO. 2021-133). The samples were preserved in an environment of −80 °C.

### Cell lines and cell culture

Human bladder urothelial carcinoma cell lines, UM-UC3 and T24, previously purchased from the Institute of Cell Biology, Chinese Academy of Sciences (Shanghai, China), were cultured in a humidified atmosphere with 5% CO_2_ at 37 °C. UM-UC3 cells were in Dulbecco’s modified Eagle’s medium (DMEM, Hyclone, USA), while T24 cells were cultured in RPMI1640 medium (Hyclone USA), both of which contained 10% fetal bovine serum (FBS, Hyclone, USA).

### Cell transfection

Short-hairpin RNA (shRNA) oligonucleotides, sh-SREBF1 and sh-ZFP64 along with the negative control shRNA, were respectively synthesized and packaged into lentivirus by GeneChem (China). The transfection was under the GeneChem transfection protocol. The shRNA sequences were exhibited in **Supplementary Table 2**.

### qRT-PCR assay

Total RNA was isolated from cells or tissues using RNAiso Plus (Takara Biotechnology, China) following the manufacturer’s protocol. Subsequently, cDNA was synthesized by reverse transcription with PrimeScript RT Master Mix (Takara Biotechnology). qRT-PCR was performed on a LightCycler 480 II system (Roche, Switzerland) using SYBR Premix Ex Taq (Takara Biotechnology) and primers. The primers were synthesized by BGI Genomics (China) and their sequences are listed in **Supplementary Table 3**. Gene expression levels were calculated via the 2^−ΔΔCt^ method, with Beta-actin serving as the internal control.

### Western blot assay

Total protein was extracted from cells or tissues using RIPA lysis buffer supplemented with 1% phenylmethylsulphonyl fluoride (PMSF). Protein concentration was determined with a bicinchoninic acid (BCA) assay kit (Beyotime, China). Equal amounts of protein were separated by SDS-PAGE and transferred onto a 0.2 μm polyvinylidene fluoride (PVDF) membrane. The membrane was then blocked with 5% non-fat milk and incubated overnight at 4°C with primary antibodies diluted at certain concentrations according to the manufacturers’ protocols. After washing, the membrane was probed with appropriate horseradish peroxidase (HRP)-conjugated secondary antibodies at 37°C for 1h. Protein bands were visualized using an EasySee Western Blot kit (Beijing Transgen Biotech, China) and a chemiluminescence imaging system (Bio-Rad, USA). Band intensities were quantified with ImageJ software. Details of the primary antibodies used are provided in **Supplementary Table 4**.

### 5-Ethynyl-2′-deoxyuridine (EdU) assay

Cell proliferation was assessed using an EdU assay kit (Beyotime, China) according to the manufacturer’s protocol. Briefly, cells were incubated with the EdU working solution (diluted 1:1000) at 37 °C under 5% CO_2_. Following labeling, cells were fixed with 4% paraformaldehyde for 15 minutes and permeabilized with 0.3% Triton X-100 for 30 minutes at room temperature. The click reaction was then performed by incubating the cells with the reaction mixture in the dark for 30 minutes at room temperature. Nuclei were subsequently counterstained with Hoechst solution for 10 minutes. Fluorescent images were captured using a fluorescence microscope (Olympus Corporation, Japan), and EdU-positive cells were quantified using ImageJ software.

### Cell viability assay (CCK-8 assay)

Cell viability was assessed using a Cell Counting Kit-8 (CCK-8; Bimake, USA). Briefly, 1.5 × 10^3^ transfected cells were seeded per well in 96-well plates. After the indicated incubation period, CCK-8 solution was added to each well according to the manufacturer’s protocol and incubated at 37 °C with 5% CO_2_ for 1 hour. Absorbance at 450 nm was then measured using a microplate reader (Model 680, Bio-Rad Laboratories).

### Wound healing assay

When transfected cells in 6-well plates reached approximately 90% confluence, a straight scratch wound was generated across the monolayer using a sterile 1000μL pipette tip. Images of the wound area were immediately acquired using an inverted microscope (EVOS XL system, AMEX1200; Life Technologies, USA). After incubating the cells in serum-free medium for 48h, images of the same regions were captured again to assess cell migration.

### Cell invasion assay

Transwell chambers (8 μm pore size; Corning, USA) were coated with Matrigel (BD, USA) diluted to 25% in serum-free medium. The lower chamber was filled with 600μL of medium containing 2% FBS, while 200μL of serum-free medium containing 3.0×10^4^ transfected cells was added to the upper chamber. After incubation for 48h under standard culture conditions, non-invading cells on the upper surface were gently removed with a cotton swab. Cells that had invaded to the lower surface were fixed and stained with 0.1% crystal violet. Stained cells were imaged using the inverted microscope mentioned above, and cell counts were obtained using ImageJ software.

### Animal experiments

All animal experiments were conducted in accordance with the institutional guidelines and were approved by the Ethics Committee for Medical Experimental Animal Welfare of China Medical University (NO. 2022274). 50 4-week-old female BALB/c-nude mice, obtained from Beijing Vital River Laboratory Animal Technology Co., Ltd., were maintained under specific pathogen-free conditions at the university’s Experimental Animal Center.

For the subcutaneous xenograft tumor model, a total of 1.0 × 10^6^ transfected UM-UC3 cells were suspended in 150 µL of serum-free DMEM mixed with 40% Matrigel, which were then injected subcutaneously into the flank of each mouse (n=5 per group). After 20 days, the mice were euthanized by cervical dislocation, after which the primary tumors were excised, measured, and weighed.

For the tail vein lung metastasis model, 1.0 × 10^5^ similarly transfected T24 cells in 150 µL of sterile PBS were injected into each mouse via the lateral tail vein (n=5 per group). After 45 days, the mice were euthanized by cervical dislocation, and the lungs were harvested and examined for metastatic nodules. The number of visible lung metastases was counted, and lung tissues were further sectioned and subjected to hematoxylin and eosin (H&E) staining for histopathological evaluation.

### Statistical analysis

The R (v 4.2.2) was utilized to conduct bioinformatics analysis. Differences analysis between groups was executed via Wilcoxon test or two-tailed Student’s t-test. Unless otherwise specified, *p* < 0.05 indicated statistical significance based on the raw *p* value.

## Results

### Identification of 119 candidate genes

**Figure 1:**
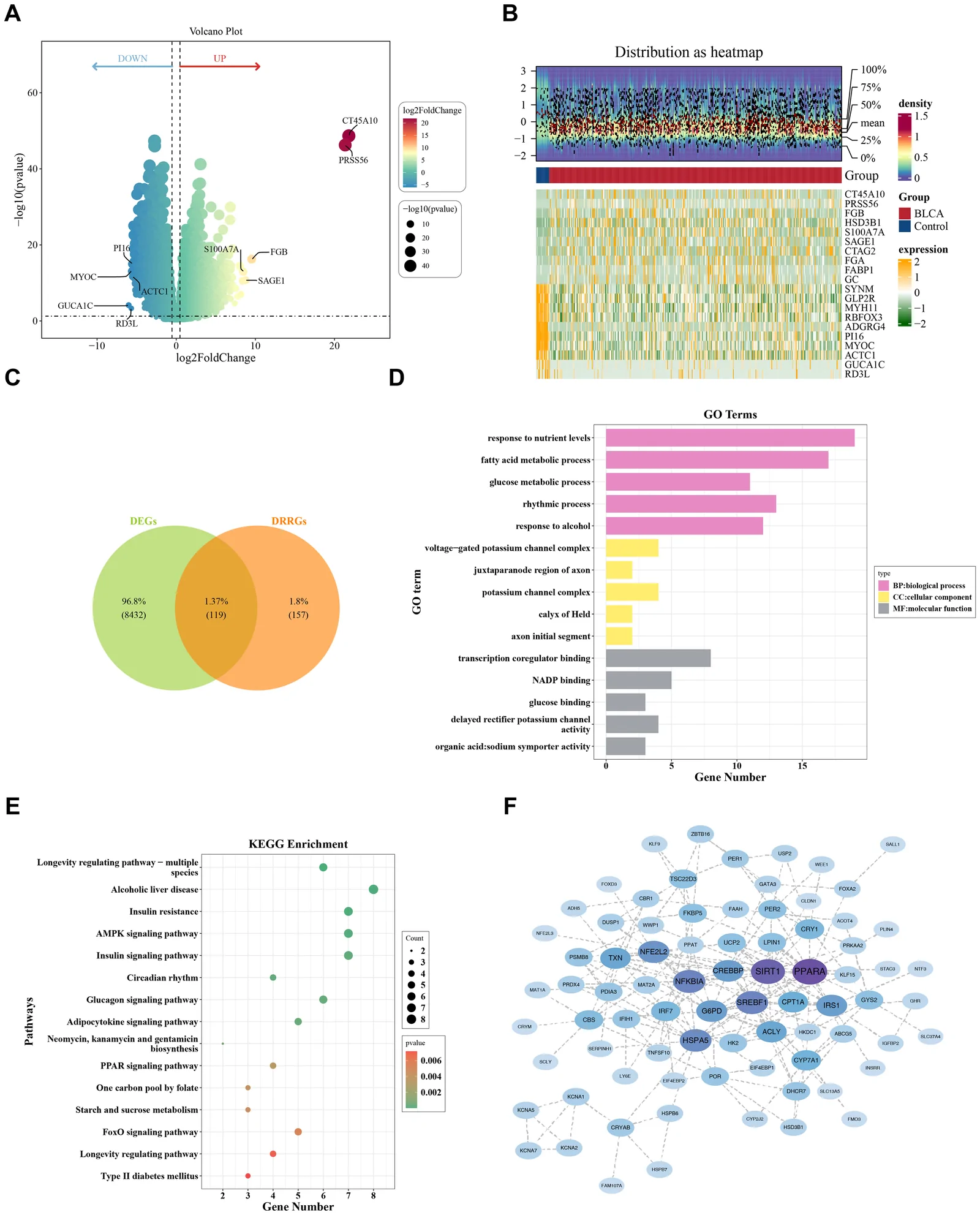
Identification of 119 DRRGs in BLCA. **(A)** DEGs between TCGA-BLCA and control samples exhibited by a volcano plot. The right side of the image represents the genes with upregulated expression, while the left side represents the genes with downregulated expression. **(B)** Heatmap showing the top 10 up-regulated and top 10 down-regulated DEGs ranked by log2FC; orange representing higher expression and green representing lower expression. **(C)** Venn diagram of 119 candidate genes obtained by overlapping DRRGs and DEGs; green indicates DEGs, orange indicates DRRGs, and the overlapping region represents 119 candidate genes. **(D, E)** Results of GO **(D)** and KEGG **(E)** enrichment analyzes for the candidate genes. **(F)** PPI network of the 119 candidate genes. Nodes represent proteins encoded by candidate genes, edges represent predicted protein interactions, and darker/larger nodes indicate higher connectivity within the network.

By differential expression analysis, there were 8,551 DEGs (5,285 up-regulated and 3,266 down-regulated) between BLCA and control samples in TCGA-BLCA (**Figures 1A, B**). To further identify genes related to dietary restriction in DEGs, 276 DRRGs were intersected with the 8,551 DEGs, yielding 119 candidate genes (**Figure 1C**). Moreover, GO functional annotation of these 119 candidate genes produced 746 results (p< 0.05), including 653 biological processes (BPs), 21 cellular components (CCs), and 72 molecular functions (MFs). Notably, GO terms included “fatty acid metabolic process” (BP), “juxtaparanode region of axon” (CC) and “glucose binding” (MF) (**Figure 1D**; **Supplementary Table 5**). Additionally, the candidate genes had significant enrichment in 39 KEGG pathways (p< 0.05), including “insulin resistance”, “alcoholic liver disease”, “AMPK signaling pathway” and so on (**Figure 1E**; **Supplementary Table 6**). The PPI network including 208 reciprocal relationships of 79 proteins was created, showing that genes such as PPARA interacted closely with many other genes within the network (**Figure 1F**). In summary, these 119 candidate genes were implicated in key biological processes and pathways, with potential roles in disease mechanisms.

### Identification of three candidate prognostic DRRGs in BLCA

**Figure 2:**
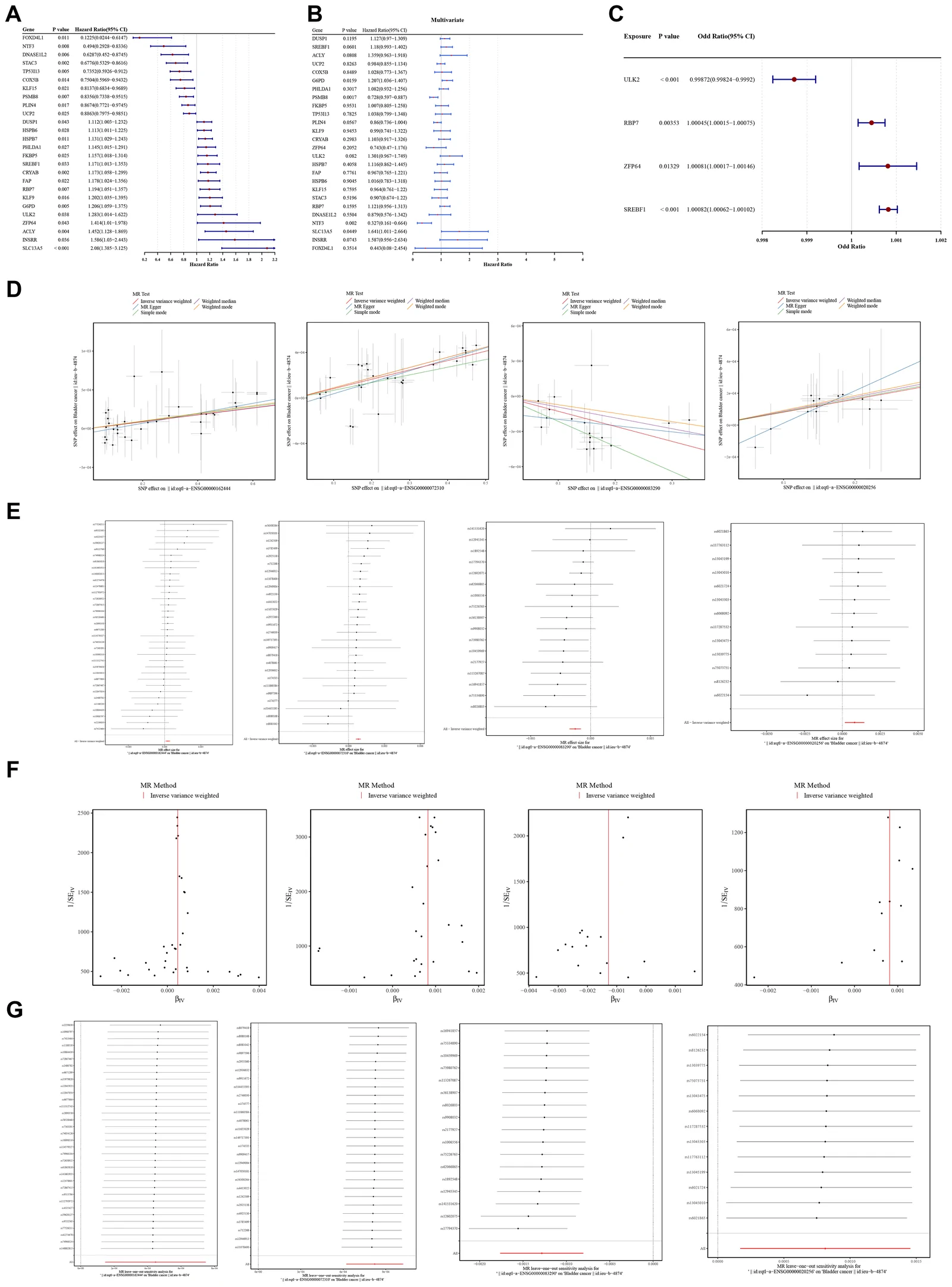
Identification of three candidate prognostic DRRGs in BLCA. **(A, B)** Forest plots showing prognostic DRRGs successively identified by univariate **(A)** and multivariate **(B)** Cox regression analyzes. Red dots indicate hazard ratios (HRs), and blue horizontal lines indicate 95% confidence intervals (CIs). **(C)** Positive MR results for candidate genes associated with BLCA. Red dots indicate odds ratios (ORs), and blue horizontal lines indicate 95% CIs. **(D)** MR scatter plots for RBP7, SREBF1, ULK2 and ZFP64. Different colored regression lines represent indicated MR methods. **(E)** Forest plots showing SNP-level MR estimates for the indicated genes. Black dots and horizontal lines indicate SNP-specific effect estimates and 95% CIs; red lines indicate the combined MR estimate. **(F)** Funnel plots assessing the symmetry of SNP effects around the IVW estimate. **(G)** Leave-one-out (LOO) sensitivity analyzes showing the influence of each SNP on the overall MR estimate. The vertical dashed line indicates the null effect where applicable.

Based on 119 candidate genes, 26 genes that were associated with prognosis were discovered using univariate Cox regression (HR ≠ 1, *p* < 0.05) and PH assumption test (*p* > 0.05) in TCGA-BLCA dataset (**Figure 2A**; **Supplementary Table 7**). Subsequently, 24 prognostic-related genes had been determined through multivariate Cox regression analysis and PH assumption test (*p* > 0.05) (**Figure 2B**; **Supplementary Table 8**).

Based on GRCh37 annotation and the TSS ±1 Mb window, the instrumental variables actually included in the main MR analysis were further reannotated. The per-SNP cis/trans annotation results are provided in **Supplementary Table 9**, and the source and cis/trans composition of instrumental variables for the candidate genes are summarized in **Supplementary Table 10**.

Based on the 24 prognosis-related genes, MR analysis was performed and four genes were found to have potential causal associations with BLCA risk. Among them, RBP7 (OR = 1.0005, 95% confidence interval [CI] = 1.0002-1.0008, p = 0.0035), ZFP64 (OR = 1.0008, 95% CI = 1.0002-1.0015, p = 0.01329), and SREBF1 (OR = 1.0008, 95% CI = 1.0006-1.0010, p< 0.001) were risk-related factors, whereas ULK2 (OR = 0.9987, 95% CI = 0.9982-0.9992, p< 0.001) showed an association in a protective direction (**Figure 2C**). The scatter plots and forest plots further supported RBP7, ZFP64, and SREBF1 as risk-related genes (positive scatter-plot slopes and MR effect estimates > 0), whereas ULK2 showed an association in a protective direction (negative scatter-plot slope and MR effect estimate< 0), with no obvious directional horizontal pleiotropy indicated (**Figures 2D, E**). In the funnel plot, the SNPs associated with 4 genes (RBP7, ZFP64, SREBF1 and ULK2) were found to be largely symmetrically distributed, suggesting that the MR analysis adheres to the principles of Mendel’s second law (**Figure 2F**). The reliability of the results of the MR study was confirmed by means of sensitivity analyzes. The IVW method showed p values greater than 0.05, suggesting that no significant heterogeneity was observed (**Supplementary Table 9**), and the *p* values for the horizontal pleiotropy test for the 4 genes were also greater than 0.05, indicating no confounding factors (**Supplementary Tables S10, S11**). A LOO sensitivity was conducted for these 4 genes, which showed that the results of the MR analysis were consistent and dependable (**Figure 2G**). The Steiger test for the 4 genes all returned true and *p* < 0.05, supporting the reasonableness of the analytical direction of the MR findings (**Supplementary Table 12**). Ultimately, SREBF1, ZFP64, and RBP7 were selected as candidate prognostic genes because MR result of ULK2 (OR< 1) was not in the same direction as univariate Cox regression analysis result (HR > 1).

To further evaluate the robustness of the MR results, we performed a sensitivity MR analysis using a more stringent threshold of p< 5 × 1–^8^ while keeping the remaining parameters unchanged; the results are summarized in **Supplementary Table 15**. Under the stringent threshold, the IVW effect directions of the four MR-positive genes identified in the main analysis remained consistent. Under this threshold, 71, 86, 52, and 36 IVW-valid SNPs were included for RBP7, SREBF1, ULK2, and ZFP64, respectively, suggesting that the stringent-threshold analysis still had a sufficient instrumental-variable basis (**Supplementary Table 16**). **Supplementary Figure 1** further shows that, under both *p* < 5 × 10–^7^ and *p* < 5 × 10–^8^ thresholds, RBP7, SREBF1, and ZFP64 were located on the OR > 1 side, whereas ULK2 was located on the OR< 1 side, supporting the directional robustness of the main MR results.

Overall, these analyzes highlighted the prognostic value of the candidate prognostic genes and their potential associations with BLCA risk. It should be noted that the OR values in the MR analysis were all close to 1, suggesting that genetically predicted expression changes in a single gene had small effect sizes on BLCA risk. Therefore, the MR results were used in this study as supportive evidence for candidate gene screening.

### Construction and validation of a risk-predicting model

**Figure 3:**
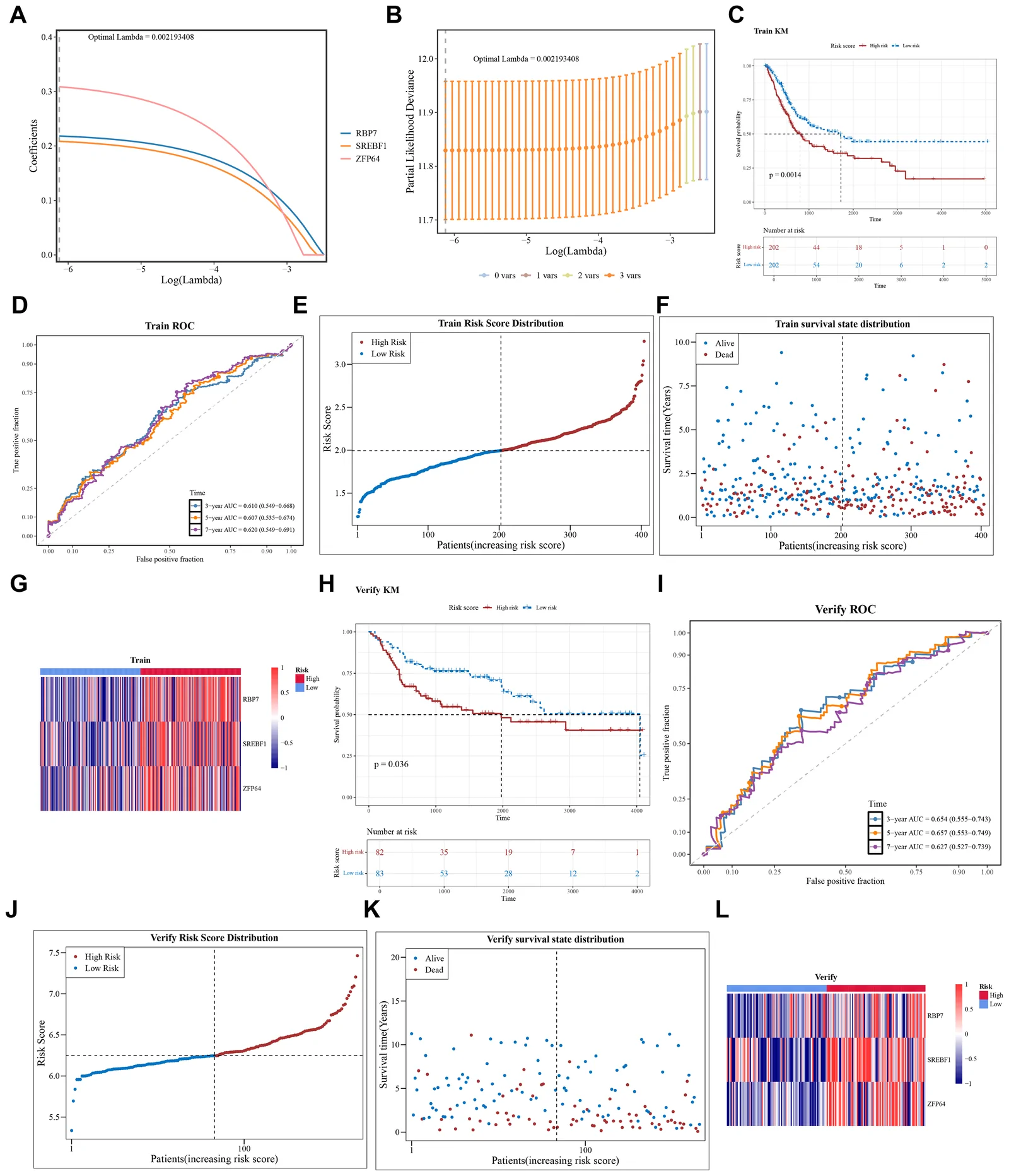
Construction and validation of the risk-predicting model. **(A)** LASSO coefficient profiles of the three prognostic genes. Different colored lines represent individual genes. **(B)** Cross-validation curve for selecting the optimal lambda value in LASSO regression; dots indicate partial likelihood deviance, and vertical error bars indicate standard errors. **(C)** K-M survival curves for the HRG and LRG patients in the training set. **(D)** ROC curves for predicting 3-, 5-, and 7-year overall survival (OS) of BLCA patients in the training set; AUC values are shown in the panel. **(E)** Distribution of risk scores in the training set; dashed lines indicate the median risk-score cutoff. **(F)** Survival status distribution in the training set. **(G)** Heatmap showing expressions of the three prognostic genes in the training set; red indicates higher expression and blue indicates lower expression. **(H, I)** K-M survival curves **(H)** and ROC curves **(I)** for the validation set. **(J–L)** Risk-score distribution **(J)**, survival status distribution **(K)** and heatmap **(L)** in the validation set, using the same color annotations as the training set.

Based on the three candidate prognostic genes, LASSO regression analysis was further performed to construct a weighted risk model. When lambda.min was 0.002193408 and log(lambda) was -6.122299, SREBF1, ZFP64, and RBP7 all retained nonzero coefficients (**Figures 3A, B**). This result indicated that none of the three genes was removed during coefficient shrinkage, and all were included in the final risk score formula. To further evaluate the internal stability of the LASSO model, 1,000 bootstrap resamples were performed. The apparent C-index of the original model was 0.4086, and the bootstrap bias-corrected C-index was 0.4211. The mean optimism was -0.0125, with a 95% CI from -0.0580 to 0.0290, suggesting that no obvious positive optimism was observed during repeated resampling (**Supplementary Figures 2A, B**). However, the absolute C-index remained low, indicating that the model’s discriminative ability should be interpreted cautiously. Calibration curve analysis showed that the predicted survival probabilities across risk score quartile groups were generally consistent with the observed survival probabilities, especially at the 3-year time point; at the 5- and 7-year time points, the intermediate-risk groups showed mild deviations, whereas the predicted values in the low- and high-risk groups remained relatively close to the observed values (**Supplementary Figures 2C, D**). These results suggested that the model had acceptable calibration but limited discrimination, and still requires further validation in independent and prospective cohorts. As a supplementary validation, the BH-adjusted p values of the three genes in the TCGA-BLCA differential expression analysis were further extracted. The results showed that RBP7 (log2FC = −1.1763, BH-adjusted *p* = 0.0063), SREBF1 (log2FC = 1.1234, BH-adjusted *p* = 2.71 × 10^-5^), and ZFP64 (log2FC = 0.6032, BH-adjusted *p* = 2.93 × 10^-5^) remained significantly differentially expressed after multiple-testing correction, which further supported the robustness of these three genes; therefore, they were defined as the final prognostic genes (**Supplementary Table 17**). Subsequently, a risk model was constructed using these three prognostic genes. According to the LASSO regression coefficients, the risk score was calculated as follows: risk score = 0.2183087 × expression (RBP7) + 0.2086101 × expression (SREBF1) + 0.3088205 × expression (ZFP64). This model stratified BLCA patients from the TCGA-BLCA dataset into 202 HRG and 202 LRG using a median risk score (1.994626). Subsequently, K-M curves showed a significant survival difference between 2 groups (p< 0.05), with lower survival rates in the HRG patients (**Figure 3C**). In the training set, the AUC values of the 3-, 5-, and 7-year ROC curves were 0.610 (95% CI: 0.549–0.668), 0.607 (95% CI: 0.535–0.674), and 0.620 (95% CI: 0.549–0.691), respectively, suggesting that the risk model had certain but limited discriminative ability (**Figure 3D**). Risk curves showing the distribution of risk scores and scatter diagrams depicting survival states demonstrated that as the sample risk score increased, the number of deaths rose significantly (**Figures 3E, F**). Additionally, the heatmap demonstrated that the three prognostic genes’ expression levels were elevated in HRG (**Figure 3G**). Similarly, 165 BLCA patients with complete survival information from GSE13507 were classified into 82 HRG and 83 LRG on the basis of the median risk score (6.245799). The main trends observed in TCGA-BLCA were partially supported in GSE13507 (**Figures 3H–L**). In the GSE13507 validation set, the AUC values of the 3-, 5-, and 7-year ROC curves were 0.654 (95% CI: 0.555–0.743), 0.657 (95% CI: 0.553–0.749), and 0.627 (95% CI: 0.527–0.739), respectively (**Figure 3I**). Overall, these results suggested that the constructed risk model had some potential for risk stratification, although its discriminative ability should still be interpreted cautiously.

### Construction of a prognostic nomogram for BLCA patients

**Figure 4:**
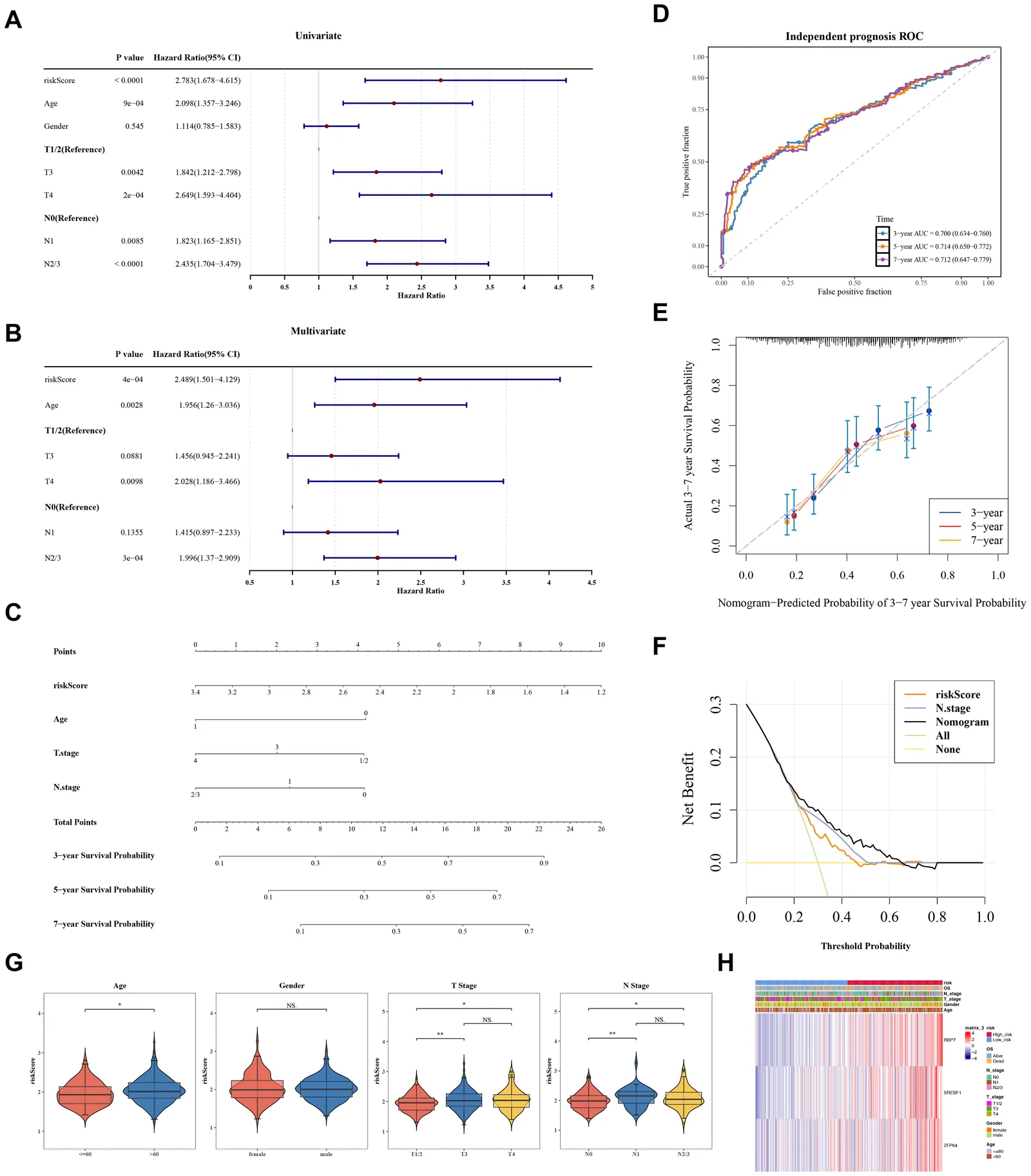
Construction of a prognostic nomogram for BLCA patients. **(A, B)** Forest plots of univariate **(A)** and multivariate **(B)** Cox regression analyzes for the risk score and the indicated clinicopathological factors; red dots indicate HRs, and blue horizontal lines indicate 95% CIs. **(C)** Nomogram integrating risk score, age, T stage, and N stage to predict 3-, 5-, and 7-year OS probabilities in TCGA-BLCA patients. Each variable corresponds to a point value on the top scale, and the sum of these points is mapped to the predicted OS probability. Age was coded as 0 for ≤60 years and 1 for >60 years; T stage was grouped as T1/2, T3, and T4; and N stage was grouped as N0, N1, and N2/3. **(D)** ROC curves evaluating the predictive performance of the nomogram for 3-, 5- and 7-year OS. **(E)** Calibration curves comparing nomogram-predicted survival probabilities with actual survival probabilities; the gray diagonal line represents ideal calibration, and colored lines represent the 3-, 5- and 7-year calibration results. **(F)** Decision curve analysis (DCA) comparing the net benefits of the risk-score model, N-stage model, and combined nomogram for predicting overall survival outcomes in patients from the TCGA-BLCA cohort across a range of threshold probabilities. **(G)** Violin plots showing risk-score differences among age, gender, T-stage and N-stage subgroups; different colors indicate the corresponding clinical subgroups. **(H)** Heatmap showing expressions of the three prognostic genes across risk and clinicopathological subgroups; red indicates higher expression and blue indicates lower expression. *p < 0.05, **p < 0.01.

Through a series of analyzes, the risk score, age, T stage, and N stage were discovered as independent prognostic factors for BLCA (**Figures 4A, B**). Consequently, a nomogram was developed using the identified independent prognostic factors. The nomogram demonstrated that elevated total points were associated with a diminished 3-, 5- and 7-year survival rate of BLCA patients (**Figure 4C**). The AUC values of the nomogram for predicting 3-, 5-, and 7-year survival outcomes were 0.700 (95% CI: 0.634–0.760), 0.714 (95% CI: 0.650–0.772), and 0.712 (95% CI: 0.647–0.779), respectively (**Figure 4D**), suggesting that it had moderate discriminative ability. The calibration curves showed that the predicted 3-, 5-, and 7-year survival probabilities were generally close to the reference line (**Figure 4E**). The DCA results showed that, in the TCGA-BLCA cohort, the combined nomogram model achieved a higher net benefit than the risk score model alone and the N-stage model across most clinically meaningful threshold probabilities, suggesting that combining the three-gene risk score with clinical predictors may provide some incremental value for prognostic assessment in BLCA patients (**Figure 4F**). Further model performance evaluation showed that the C-index of the risk model was 0.668, which also supported a certain degree of discriminative ability. Calibration-related indicators showed that the 3-, 5-, and 7-year Brier scores were 0.215, 0.229, and 0.237, respectively, and the IPCW-corrected Brier scores were 0.217, 0.204, and 0.218, respectively. The calibration slopes were 0.893, 0.850, and 0.816, and the calibration intercepts were -0.467, -0.649, and -0.726, respectively, suggesting that some calibration bias existed between the model predictions and observed outcomes and that the bias slightly increased with longer prediction time. Therefore, this independent prognostic model was interpreted as having moderate predictive ability and potential value for risk stratification, rather than as a high-performance prediction model that could be directly used for clinical decision-making (**Supplementary Table 18**). The results of the correlation analysis between clinical characteristics and risk scores demonstrated significant disparities in risk scores with respect to age, T stage, and N stage, as well as elevated expression levels of prognostic genes in the HRG (**Figures 4G, H**; **Supplementary Table 19**).

### Differences of signaling pathways, immune cells and immune checkpoints between HRG and LRG

**Figure 5:**
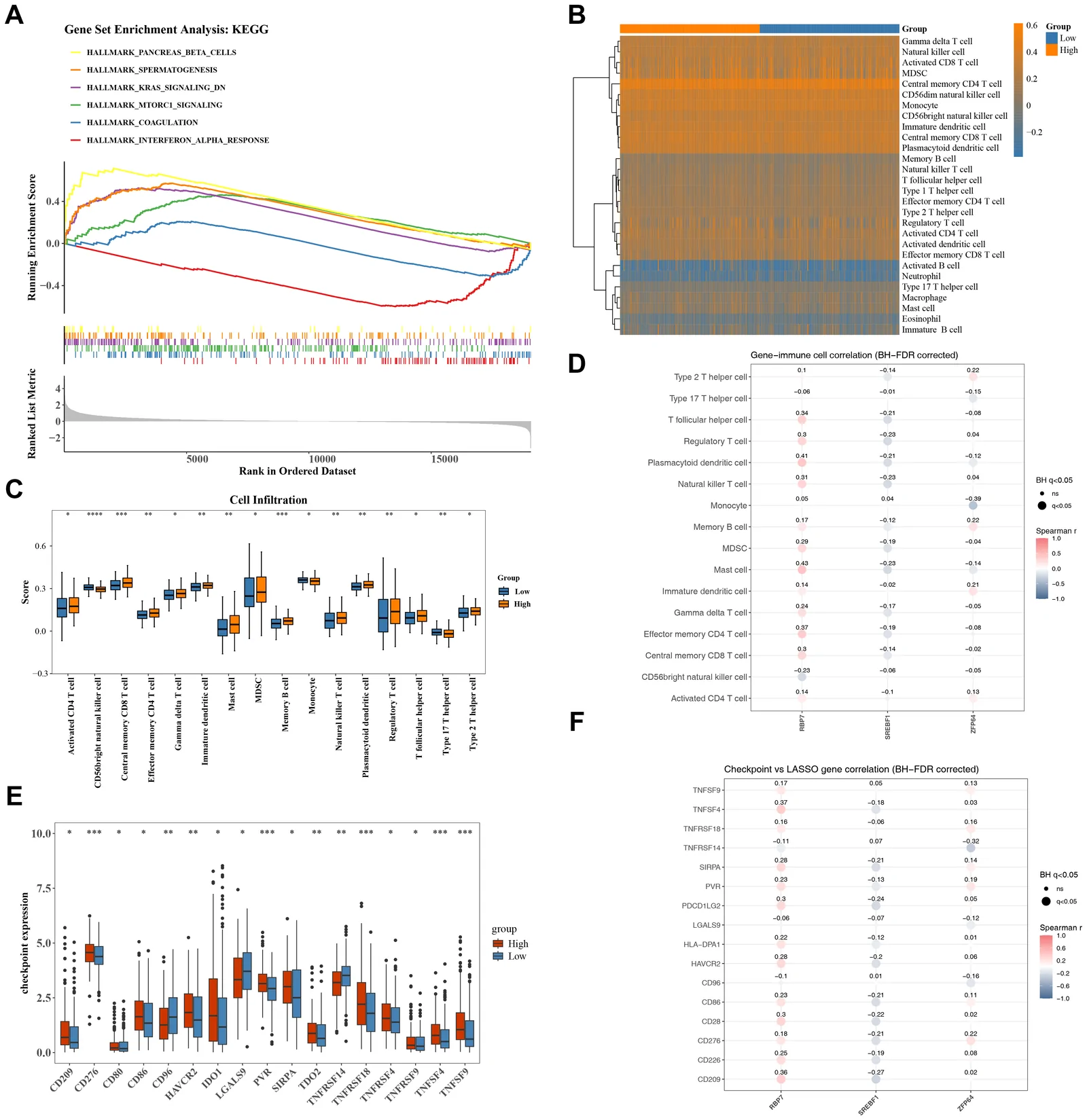
Differences in signaling pathways, immune cell infiltration and immune checkpoint expression between HRG and LRG. **(A)** Partial visualization of GSEA analysis results showing hallmark pathways enriched in HRG and LRG. **(B)** Heatmap showing single-sample GSEA (ssGSEA)-derived enrichment scores of differentially infiltrated immune cells; orange indicates higher enrichment scores and blue indicates lower enrichment scores. **(C)** Boxplots comparing enrichment scores of the indicated immune cells between HRG and LRG. **(D)** Correlation matrix showing associations between prognostic gene expression and immune cell infiltration. **(E)** Boxplots comparing expression levels of checkpoint genes between HRG and LRG. **(F)** Correlation matrix showing associations between the expressions of prognostic genes and immune checkpoint genes; red indicates positive correlation, blue indicates negative correlation, and larger dots indicate stronger statistical significance. *p < 0.05, **p< 0.01, ***p < 0.001 and ****p< 0.0001.

GSEA revealed significant differences between the HRG and LRG in 12 pathways like epithelial-mesenchymal transition (EMT), MTORC1 signaling and G2M checkpoint (**Figure 5A**; **Supplementary Table 20**). Significant differences were found in 16 immune cells (like mast cells and regulatory T cells) in the immune infiltration analysis between 2 groups (*p<* 0.05) (**Figures 5B, C**). The results in **Figure 5D** showed that the correlation coefficient between RBP7 and mast cells was relatively high, but still indicated a low-to-moderate positive correlation (cor = 0.43, BH-adjusted *p* < 0.05); ZFP64 showed a low-to-moderate negative correlation with monocytes (cor = -0.38, BH-adjusted *p* < 0.05). Differential expression abundance of 17 immune checkpoint genes was observed between the HRG and LRG, and almost all these genes showed higher expression in the HRG, suggesting that the HRG may have an immune-related state characterized by higher immune checkpoint expression (**Figure 5E**). In addition, the differential immune checkpoint CD209 showed a statistically significant but limited positive correlation with RBP7 (cor = 0.38, BH-adjusted *p* < 0.05) (**Figure 5F**). Overall, these results suggested that BLCA patients at different risk levels had certain differences in molecular pathways, immune cell infiltration, and immune checkpoint expression. The correlation analysis further provided clues for a potential link between prognostic DRRGs and immune microenvironment features, but the overall correlation coefficients were in the low-to-moderate range; therefore, further functional experiments are still required to validate their biological significance.

### Differences of mutated genes between HRG and LRG

**Figure 6:**
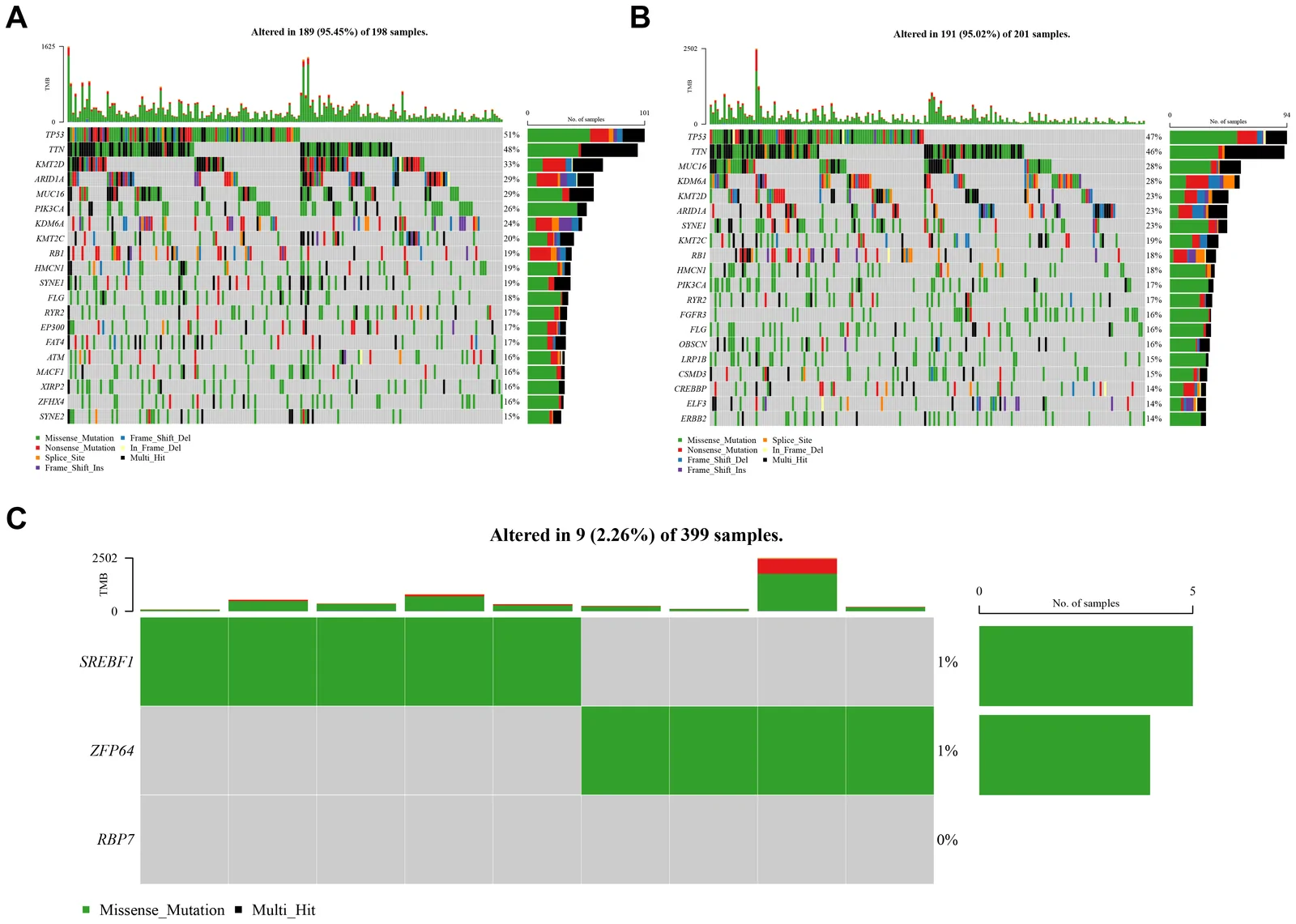
Differences in mutated genes between HRG and LRG. **(A, B)** Waterfall plots showing the top mutated genes in HRG **(A)** and LRG **(B)**. Each column represents one sample and each row represents one gene. The upper bar plot shows tumor mutational burden (TMB), and the right bar plot shows mutation frequency for each gene. **(C)** Waterfall plot showing somatic mutation status of RBP7, SREBF1 and ZFP64. Gray indicates no detected mutation, and colored blocks indicate mutation types as defined in the legend.

In the HRG, top three genes with the highest mutation frequencies were TP53 (51%), TTN (48%) and KMT2D (33%). In contrast, in the LRG, the top genes were TP53 (47%), TTN (46%) and MUC16 (28%). The predominant types of mutations observed included missense mutations, nonsense mutation, splice site and so on (**Figures 6A, B**). Moreover, prognostic genes occurred more often missense mutation (**Figure 6C**). In conclusion, these findings suggest a potential impact of mutations on prognosis.

### Differences in immunotherapy-related predictive indicators and drug sensitivity between the HRG and LRG

**Figure 7:**
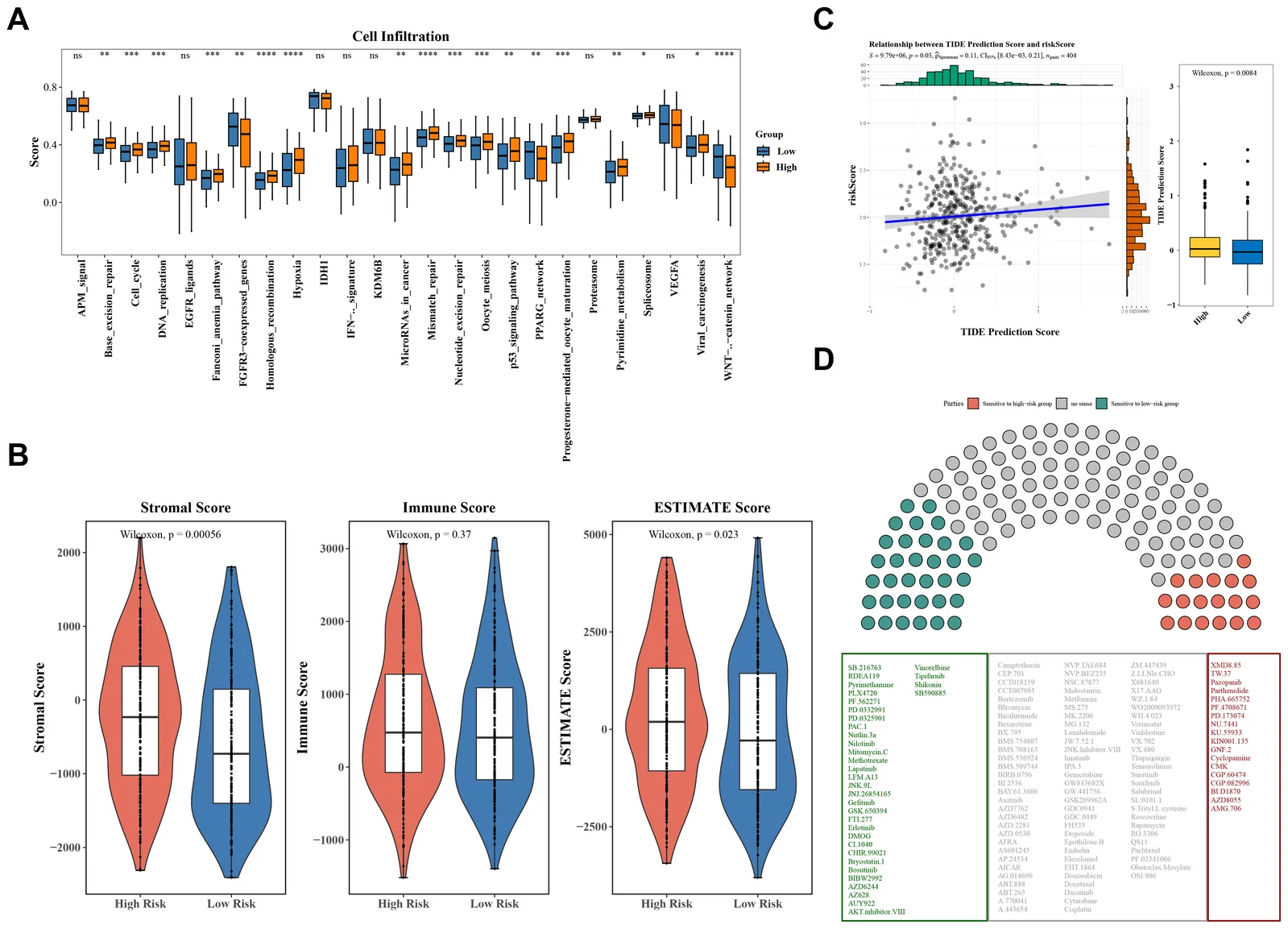
Differences in immunotherapy-related predictive indicators and predicted drug sensitivities between HRG and LRG. **(A)** Boxplots showing ssGSEA-derived activity scores of immunotherapy-related predictive pathways in HRG and LRG. **(B)** Violin plots comparing stromal score, immune score and Estimation of Stromal and Immune cells in Malignant Tumor tissues using Expression data (ESTIMATE) score between HRG and LRG. **(C)** Correlation between TIDE prediction score and the risk score; gray points represent individual patients, the blue line represents the fitted regression line, and the gray shaded area represents the confidence interval; the marginal histograms show the score distributions. The adjacent boxplot compares TIDE prediction scores between HRG and LRG. **(D)** Drug sensitivity analysis based on IC50 values; red circles indicate drugs predicted to be more sensitive in HRG, green circles indicate drugs predicted to be more sensitive in LRG, and gray circles indicate no obvious risk-group preference. ns indicates non-significant, *p < 0.05, **p < 0.01, ***p < 0.001 and ****p < 0.0001.

We further detected significant differences between the two groups in 18 immunotherapy-related predictive pathways, such as the cell cycle and hypoxia (*p* < 0.05) (**Figure 7A**). Meanwhile, as shown in **Figure 7B**, the stromal scores and ESTIMATE scores differed significantly between HRG and LRG *(p* < 0.05), while the immune scores exhibited no statistical significance *(p* = 0.37). The TIDE scores of the HRG and LRG differed significantly (*p* = 0.0084) and had some positive correlation, but the correlation was not strong (cor = 0.11, BH-adjusted *p* = 0.03) (**Figure 7C**). The higher TIDE score in the HRG suggested that this group may have immune-evasion-related transcriptional features; however, because the correlation between the risk score and TIDE prediction score was very weak, its relationship with prognosis should still be interpreted cautiously. Additionally, a total of 52 drugs were predicted to have significantly different IC50 values between HRG and LRG, including vinorelbine, shikonin and so on (**Figure 7D**). Collectively, these findings highlighted distinct immunotherapy-related predictive indicators and drug sensitivity profiles in the HRG and LRG, which may influence treatment responses in the two risk groups.

### T cells were selected as the potential key cell type related to prognostic DRRG expression

**Figure 8:**
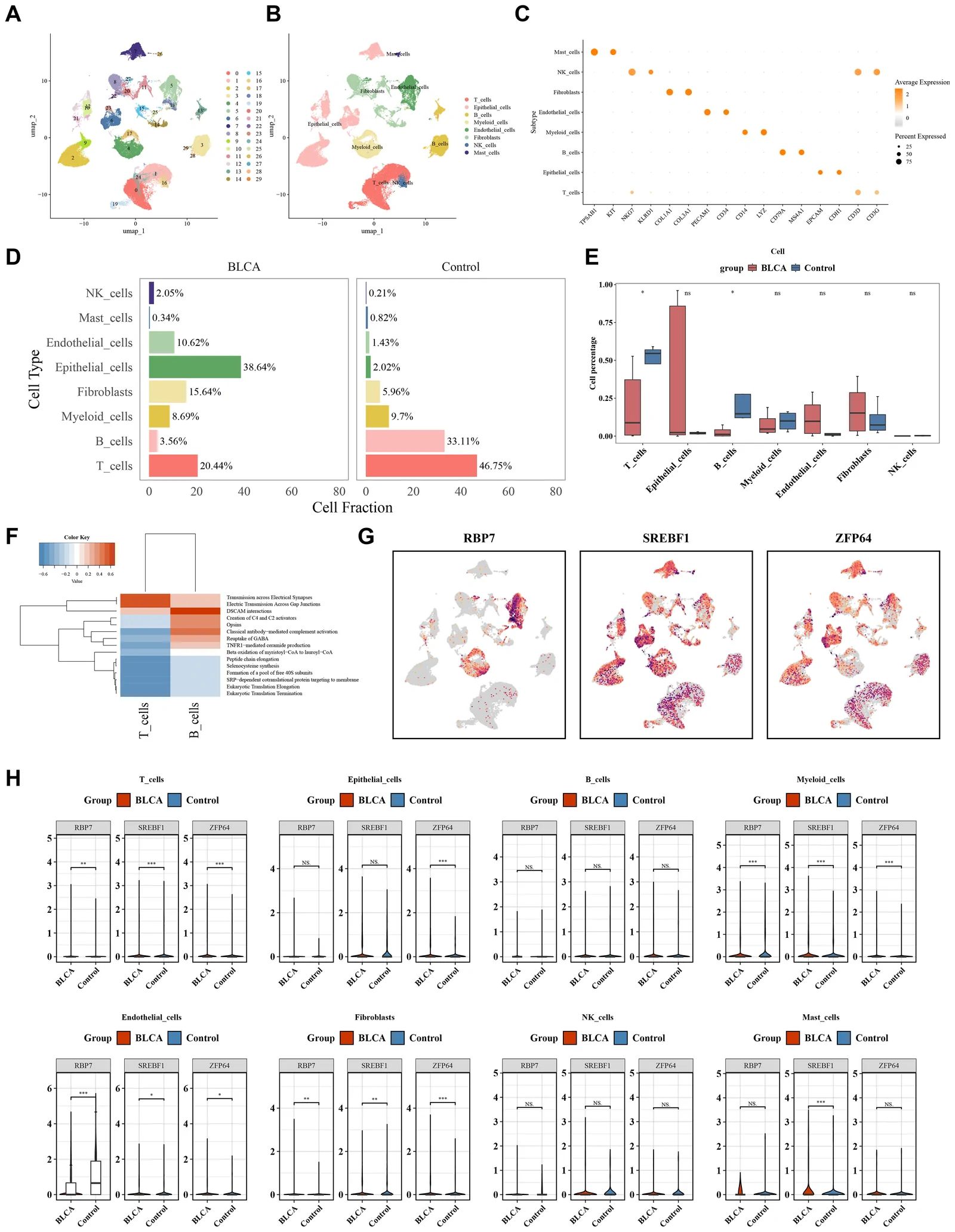
Identification of T cells as a DRRG-associated cell type by scRNA-seq analysis. **(A)** UMAP plot showing 30 cell clusters after QC and dimensionality reduction; each color represents a distinct cluster. **(B)** UMAP plot showing annotation of eight major cell types. **(C)** Dot plot showing marker-gene expression across the eight cell types; dot color indicates average expression, and dot size indicates the percentage of cells expressing the marker. **(D)** Bar plots showing proportions of the eight cell types in BLCA and control samples. **(E)** Boxplots comparing cell-type proportions between BLCA and control samples. **(F)** Functional enrichment heatmap for T cells and B cells; red indicates relatively higher pathway activity and blue indicates relatively lower pathway activity. **(G)** UMAP feature plots showing the distribution of cells expressing RBP7, SREBF1 and ZFP64; gray indicates low or undetectable expression, and warmer colors indicate higher expression. **(H)** Violin plots comparing expression of the three prognostic DRRGs in each cell type between BLCA and control samples. NS indicates non-significant, *p < 0.05, **p < 0.01 and ***p < 0.001.

The results before and after QC were shown in **Supplementary Figures 3A, B**. After standard data processing, we selected 2,000 highly variable genes for subsequent analysis, of which the top 5 genes exhibiting the most pronounced intercellular expression changes were labeled (**Supplementary Figure 3C**). Following this, the samples showed no clear boundaries (**Supplementary Figure 3D**). The top 30 PCs were chosen for further analysis (**Supplementary Figures 3E, F**). Then, a total of 30 different cell clusters were identified, which were subsequently annotated to eight cell types, namely Natural Killer (NK) cells, mast cells, fibroblasts, endothelial cells, epithelial cells, myeloid cells, B cells and T cells (**Figures 8A, B**). The bubble plot demonstrated that the marker genes exhibited high specificity (**Figure 8C**).

To verify whether the selection of resolution = 0.5 influenced the main clustering conclusions, we further performed clustering robustness testing at different resolutions. The results showed that resolutions of 0.1, 0.3, 0.5, 0.7, and 1.0 yielded 13, 24, 30, 41, and 46 cell clusters, respectively, with the corresponding UMAP results provided in 02.UMAP_multi_resolution. Compared with the main analysis at resolution = 0.5, the ARI for resolution = 0.3 was 0.936, suggesting highly consistent clustering structures, and the ARI for resolution = 0.7 was 0.855, indicating that the main clustering framework remained stable. In contrast, the ARI for resolution = 0.1 was 0.667, suggesting some overaggregation, whereas the ARI for resolution = 1.0 was 0.734, suggesting potential oversplitting. These results indicated that resolution = 0.5 fell within a relatively robust resolution range and that the identification of major cell types did not depend on an accidental choice of a single clustering parameter (**Supplementary Figure 4**). We further applied CopyKAT to infer the malignant identity of epithelial cells based on copy-number variation features. The results showed that 26,035 epithelial cells were detected in BLCA samples, of which 5,469 were classified as aneuploid, corresponding to an aneuploid proportion of 21%; in contrast, 484 epithelial cells were detected in control samples, and no aneuploid cells were identified, corresponding to an aneuploid proportion of 0% (**Supplementary Table 20**). This result suggested that BLCA-derived epithelial cells contained identifiable aneuploid malignant epithelial components, whereas control-derived epithelial cells mainly exhibited diploid features. It also suggested a certain degree of heterogeneity within tumor-derived epithelial-cell populations.

**Figure 8D** showed the proportion of eight cell types between BLCA and control groups. Concretely, the proportion of epithelial cells was greatest in BLCA group. Furthermore, B cells and T cells differed significantly between the BLCA and control groups and were selected as the differential cell types (**Figure 8E**). The analysis of functional enrichment revealed that B cells were mainly enriched to DSCAM interactions, while T cells were mainly enriched to electrical transmission across gap junctions (**Figure 8F**). In addition, the UMAP plots exhibited more cells expressing SREBF1 and ZFP64 (**Figure 8G**). Ultimately, T cells, a differential cell type, which exhibited differential expressions of all prognostic genes between BLCA and control samples were identified as potential key cells related to prognostic DRRG expression (*p* < 0.05) (**Figure 8H**). To further clarify the internal heterogeneity of T cells, T cells were extracted and re-clustered and subcluster annotation was performed. Based on the expression patterns of classical T-cell functional markers, different T-cell subclusters showed distinct functional-state characteristics. The bubble plot showed that CD3D was broadly expressed in most T-cell subclusters, supporting their T-cell identity; CD4 and CD8A were relatively enriched in different subclusters, suggesting the presence of CD4+ and CD8+ T-cell-related subclusters; FOXP3 was expressed in some subclusters, suggesting regulatory T-cell-related features; and immune checkpoint/exhaustion-associated markers such as PDCD1, CTLA4, and HAVCR2 were expressed in some T-cell subclusters, suggesting that T cells might exhibit certain functional exhaustion- or immunosuppression-related states (**Supplementary Figure 5A**). Further analysis of the expression distribution of the three prognostic DRRGs in T-cell subclusters showed that SREBF1 and ZFP64 were detectable in multiple T-cell subclusters, whereas RBP7 had a relatively limited expression range (**Supplementary Figures 5B, C**). These results suggested that T cells were not a homogeneous population but contained subclusters with different regulatory, effector, and exhaustion-related features; the expression distribution of prognostic DRRGs also differed across T-cell subclusters. These findings provided more specific single-cell-level evidence for prioritizing the relationship between T cells and prognostic DRRG expression.

### T cells communicated with a variety of cell types

**Figure 9:**
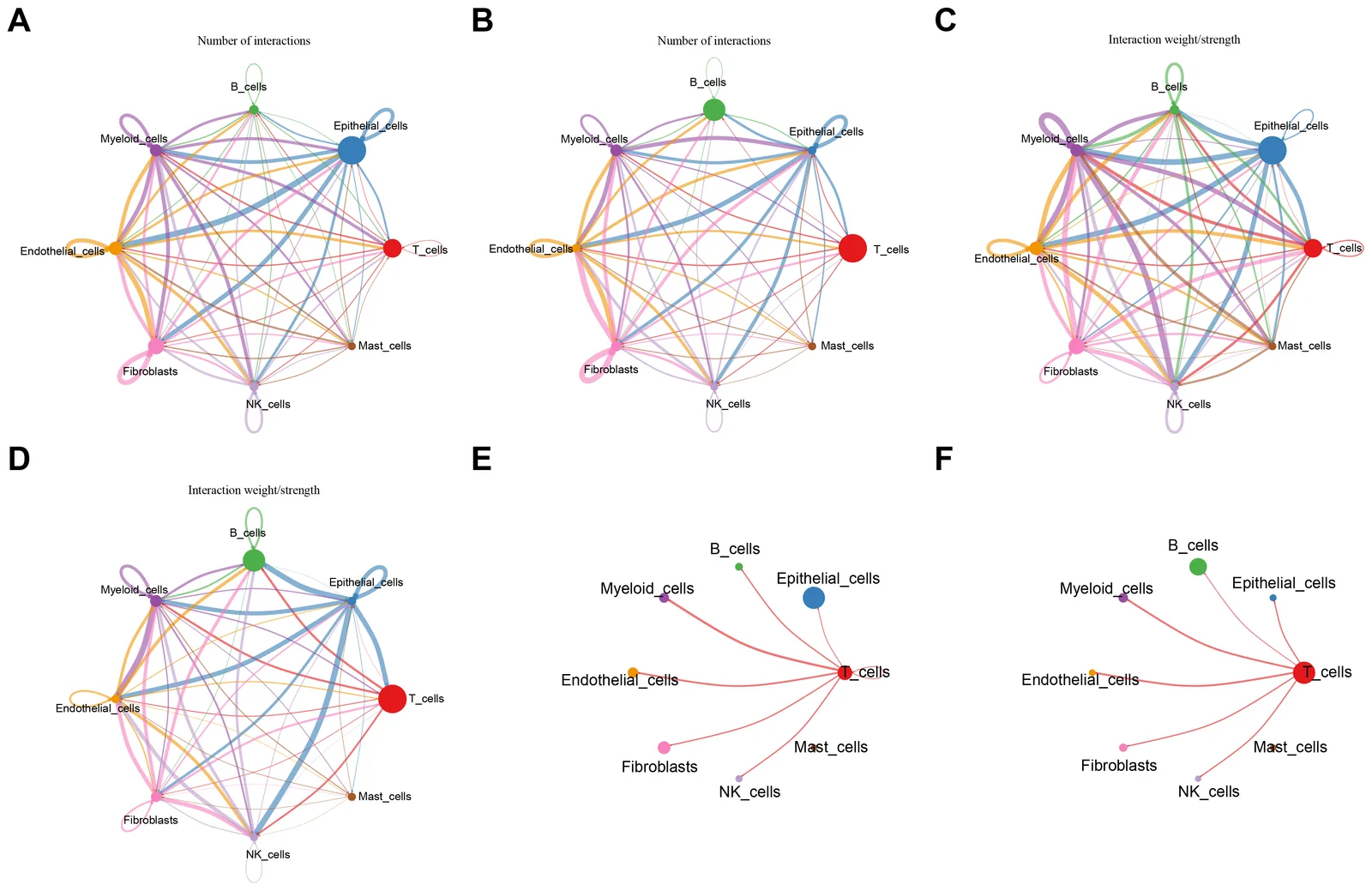
Cell-cell communication patterns involving T cells. **(A, B)** Numbers of predicted interactions among the eight cell types in BLCA **(A)** and control **(B)** samples. Node colors represent cell types, node size reflects the total number of interactions associated with each cell type, and edge width reflects the number of interactions between cell types. **(C, D)** Strengths of predicted interactions among the eight cell types in BLCA **(C)** and control **(D)** samples. Thicker edges indicate stronger predicted communication. **(E, F)** T-cell-centered interaction networks in BLCA **(E)** and control **(F)** samples. Red edges indicate predicted interactions involving T cells, and node size represents the relative interaction contribution of each cell type.

We conducted an analysis of intercellular communication networks between different cell types in the BLCA and control groups. Particularly, T cells had communication relationships with NK cells, fibroblasts, epithelial cells, myeloid cells, B cells, and endothelial cells (**Figures 9A–D**).

### T cells differentiated over time

**Figure 10:**
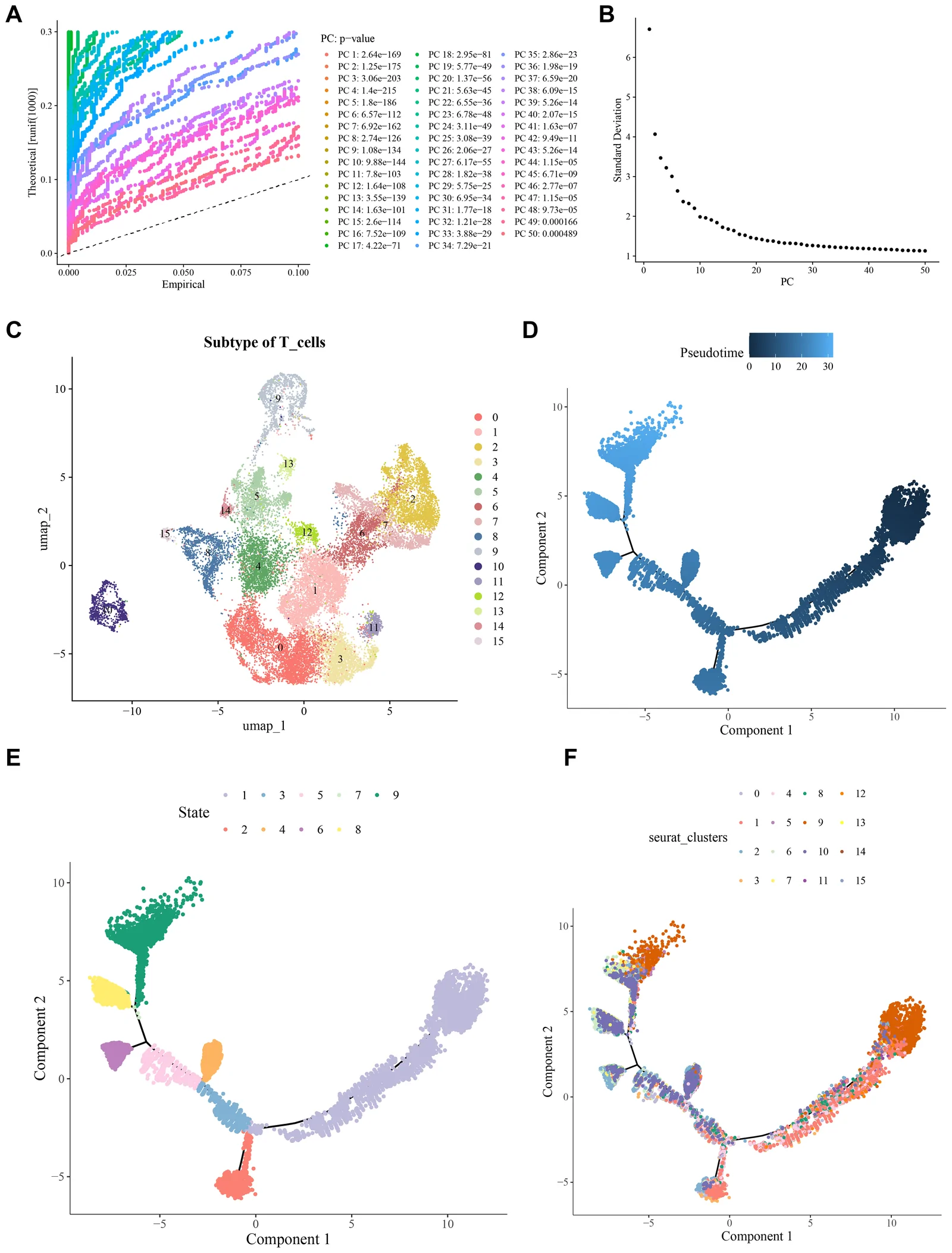
Heterogeneity and pseudotime trajectory of T cells. **(A)** Statistically significant PCs for T cells identified by JackStrawPlot; different colors represent individual PCs. **(B)** ElbowPlot selecting the number of PCs for downstream clustering; the curve begins to flatten around PC 20. C UMAP plot showing 16 T cell subclusters, with each color representing a distinct subcluster. **(D)** Pseudotime trajectory of T cells inferred by Monocle. The color gradient from dark blue to light blue indicates increasing pseudotime. **(E)** Mapping of nine cell states onto the pseudotime trajectory; different colors represent different inferred states. **(F)** Mapping of the 16 T-cell subclusters onto the pseudotime trajectory. Different colors represent different Seurat clusters.

In GSE222315, secondary clustering based on T cells was performed, reclassifying the T cells into 16 distinct subpopulations (**Figures 10A–C**). In addition, in the results of the pseudo-time trajectory inference analysis, the trajectories of T cells were shown in **Figures 10D–F**. Cells differentiate from right to left over time. T cells were found to include a total of 9 different states of differentiation and 16 subtypes. For T cells, state 1 was the earliest stage of its development, while state 9 was the latest stage of its development. The subtype 12 of T cells was present at both pre- and post-differentiation stages of cellular differentiation.

### SREBF1 and ZFP64 were overexpressed in BLCA

**Figure 11:**
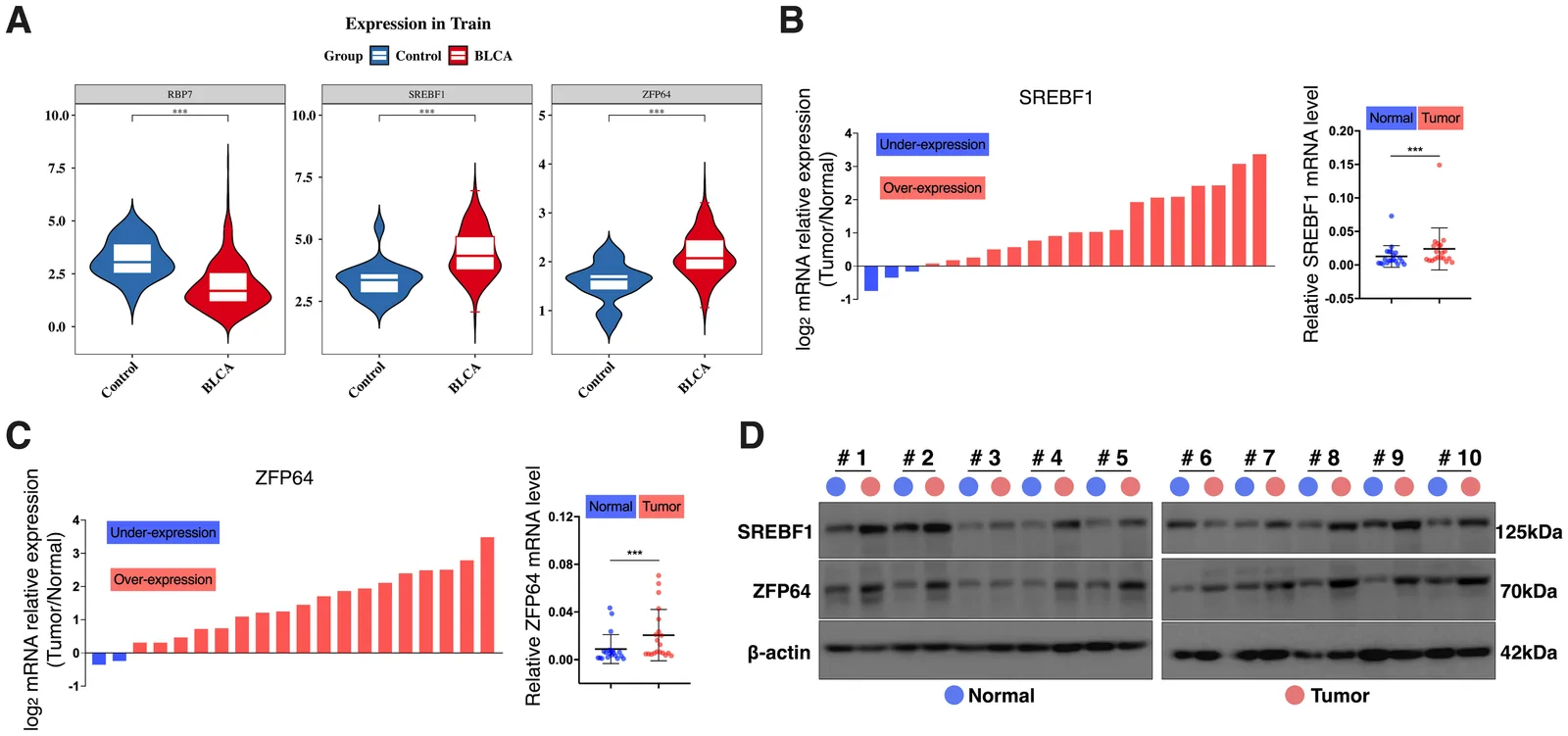
Expression validation of SREBF1 and ZFP64 in BLCA. **(A)** Violin plots comparing RBP7, SREBF1 and ZFP64 expression between TCGA-BLCA tumor samples and control samples. **(B, C)** qRT-PCR assay of SREBF1 **(B)** and ZFP64 **(C)** mRNA expression in paired BLCA tumor and adjacent normal tissues. In the bar plots, red indicates overexpression in tumor tissue relative to normal tissue and blue indicates under-expression. In the paired scatter plots, red indicates tumor samples and blue indicates normal samples. **(D)** Protein levels of SREBF1 and ZFP64 in BLCA and control samples detected by western blot assay. Wilcoxon test was used. ***p < 0.001.

The expression analysis results of the three prognostic genes indicated that SREBF1 and ZFP64 showed significantly elevated expression levels in BLCA group (*p* < 0.05), while RBP7 showed significantly elevated expression levels in the control group (*p* < 0.05) (**Figure 11A**). With a higher probability of being therapeutic targets, SREBF1 and ZFP64 were selected for further experimental validations. qRT-PCR results demonstrated both these two molecules were overexpressed at transcriptomic level in BLCA and their protein overexpression was then observed by western blot assay (**Figures 11B–D**).

### Knockdown of SREBF1 and ZFP64 suppressed malignant phenotypes of BLCA cells

**Figure 12:**
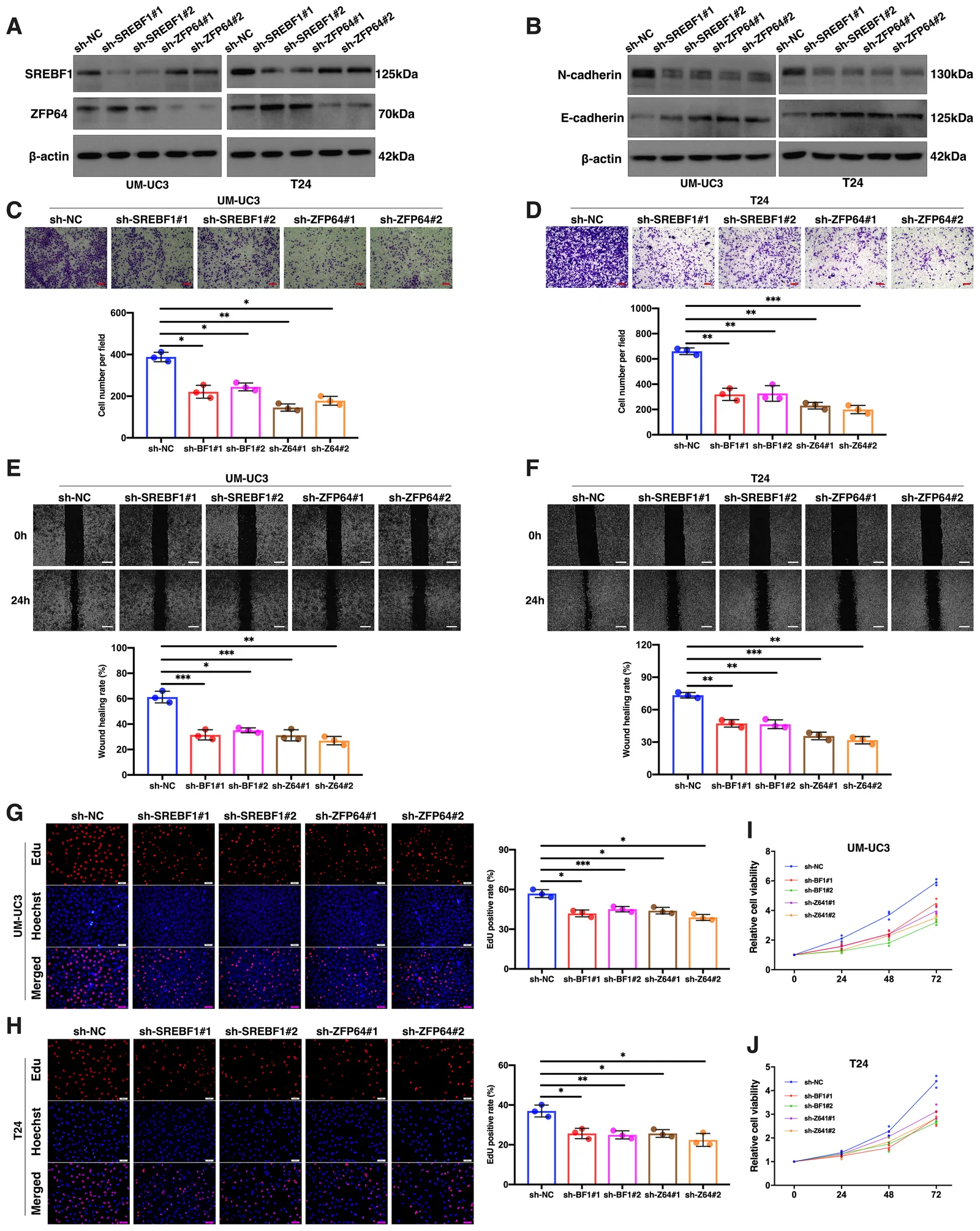
Knockdown of SREBF1 and ZFP64 suppressed malignant phenotypes of BLCA cells *in vitro*. **(A)** Western blot analysis showing knockdown efficiency of shRNAs targeting SREBF1 or ZFP64 in UM-UC3 and T24 cells; sh-NC indicates negative-control shRNA. **(B)** Western blot analysis showing changes in EMT-related proteins, N-cadherin and E-cadherin, after SREBF1 or ZFP64 knockdown. **(C, D)** Representative Transwell invasion images and quantification in UM-UC3 **(C)** and T24 **(D)** cells. **(E, F)** Representative wound-healing images and quantification in UM-UC3 **(E)** and T24 **(F)** cells. **(G, H)** Representative EdU staining images and quantification in UM-UC3 **(G)** and T24 **(H)** cells; red indicates EdU-positive proliferating cells and blue indicates Hoechst-stained nuclei. **(I, J)** CCK-8 assays showing cell viability in UM-UC3 **(I)** and T24 **(J)** cells. Scale bar = 50 μm. Independent triplicates were performed for each experiment. Data are presented as mean ± standard error of the mean (SEM). Two-tailed Student’s t-test was performed. *p < 0.05, **p < 0.01 and ***p < 0.001.

**Figure 13:**
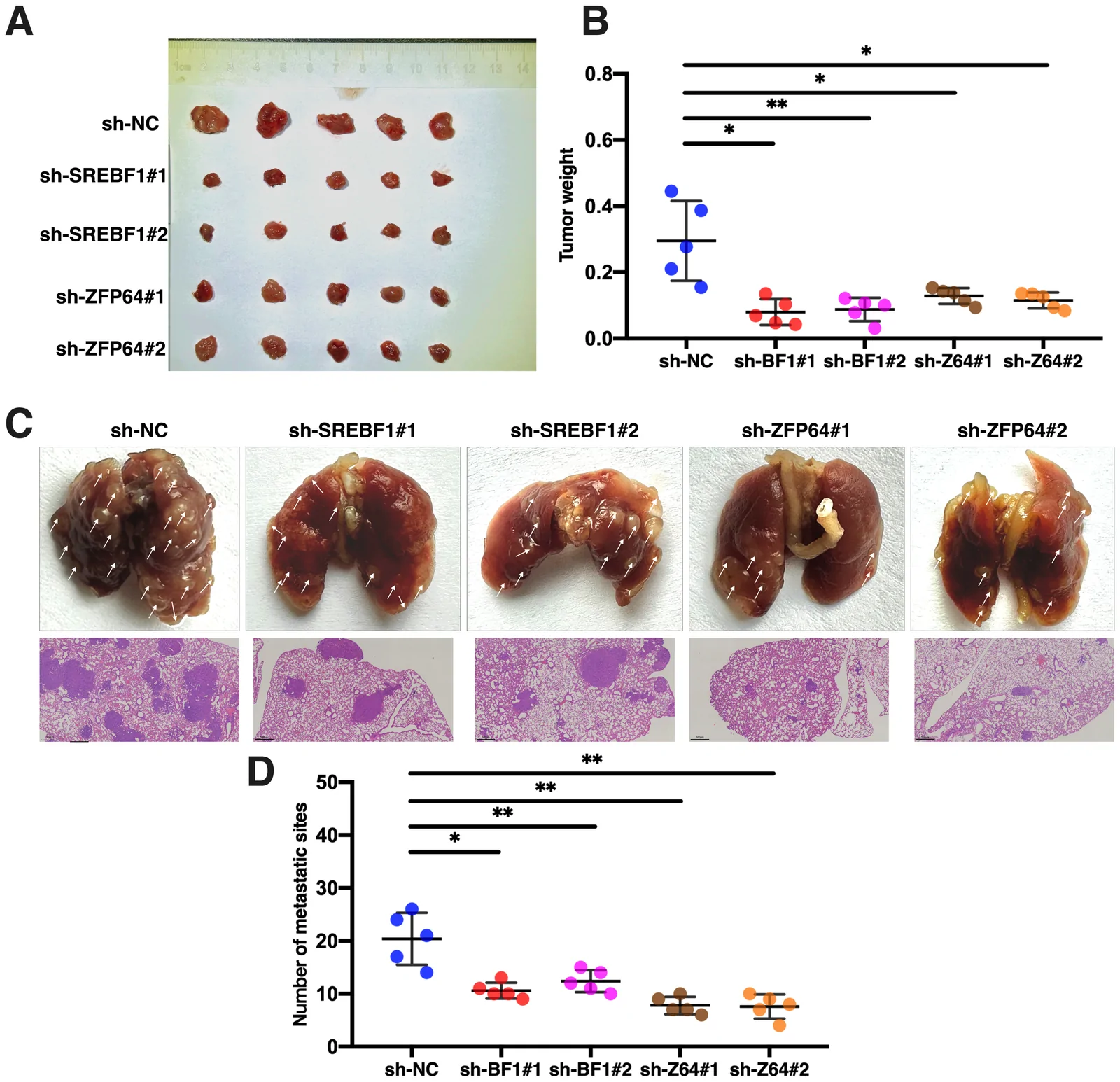
Knockdown of SREBF1 and ZFP64 suppressed BLCA cell growth and metastasis *in vivo*. **(A)** Representative subcutaneous tumors excised from BALB/c-nude mice injected with UM-UC3 cells transfected with sh-NC, sh-SREBF1 or sh-ZFP64. **(B)** Quantification of subcutaneous tumor weight; each dot represents one mouse (n = 5 per group). **(C)** Representative gross lung specimens and H&E-stained lung sections from BALB/c-nude mice injected via tail vein with T24 cells after the indicated treatments; white arrows indicate visible lung metastatic nodules. **(D)** Quantification of lung metastatic sites; each dot represents one mouse. Data are presented as mean ± SEM. Two-tailed Student’s t-test was performed. *p < 0.05 and **p< 0.01.

To further explore the biological functions of the target genes, we first respectively knocked down SREBF1 and ZFP64 in two BLCA cell lines, UM-UC3 and T24 (**Figure 12A**). According to western blot results, each knockdown led to a decrease in N-cadherin abundance, while E-cadherin exhibited an overexpression, which suggested an inhibition of EMT (**Figure 12B**). Subsequent functional experiments were performed. We observed that the depletion of either SREBF1 or ZFP64 could significantly impair the invasiveness, migration, proliferation and viability of BLCA cells *in vitro* (**Figures 12C–J**). Then, we constructed a subcutaneous xenograft tumor model to assess the impact of these two molecules on BLCA cell growth *in vivo*, of which the result showed reductions in tumor volumes and weights by the knockdown of SREBF1 or ZFP64 (**Figures 13A, B**). Moreover, SREBF1 insufficiency was proved to suppress BLCA cell metastasis *in vivo*, and so was ZFP64 knockdown (**Figures 13C, D**).

## Discussion

DR, a term referring to reduced intake of calories or specific nutrients without malnutrition, has long been considered an important tumor-suppressive intervention with direct actions on cancer cells and the most potent non-pharmacological intervention against malignancies (53). The crucial role of dietary patterns in regulating tumorigenesis and cancer therapy has been demonstrated by numerous studies (54, 55). In 2025, a study reported a positive correlation between BLCA risk and a dietary pattern rich in highly processed and high-calorie foods (56). However, as DR-related molecular factors, changes in the expression or activity of DRRGs still need to be comprehensively evaluated.

In the present study, using transcriptome data and clinical information from multiple public datasets, we obtained three prognosis-related DRRGs for BLCA, RBP7, SREBF1 and ZFP64 by univariate and multivariate COX regression analyzes as well as MR analysis. A risk prediction model with moderate performance was then constructed based on the expression of these target genes and was validated as an independent prognostic factor. Meanwhile, integrating the risk score with three other independent variables (age, T stage, and N stage) yields a prognostic nomogram for BLCA patients with moderate predictive ability and potential risk-stratification value. More importantly, BLCA patients in LRG and HRG were detected with differences in signaling pathway enrichments, immune cells, immune checkpoints, genetic mutations. immunotherapy-related predictive indicators and drug sensitivities, which reveals the molecular mechanisms for BLCA progression to an extent and potentially provides individualized therapeutic strategies for patients of various risks.

RBP7 belongs to the cellular retinol-binding protein (CRBP) family, which regulates vitamin A stability and metabolism (57). It should be noted that RBP7 is not reported for the first time in BLCA prognostic research. Previous studies have included RBP7 in an immune-related gene prognostic signature and validated its association with prognostic stratification of BLCA patients in TCGA, GSE13507, and other cohorts (58, 59). In addition, RBP7 has also been reported to be potentially regulated by EBF1 transcription and to participate in BLCA bone metastasis by affecting Th2 cell-related immune processes and oocyte meiosis-related pathways (60). Therefore, in this study, RBP7 should be understood as a candidate prognostic variable reidentified in the context of DR-related transcriptional features, rather than as a completely novel BLCA gene or a validated DR-specific functional gene. SREBF1, usually overexpressed in cancers, encodes a transcription factor regulating fatty acid metabolism (61). Activated by FGFR3 a PI3K-mTORC1-dependent manner, SREBF1 uplifts the expression of a key lipogenic enzyme, stearoyl CoA desaturase 1 (SCD1) that strengthens malignant characteristics of BLCA cells (62). ZFP64 encodes a transcription factor containing a zinc finger domain, classifying it within the zinc finger protein family (63). Directly interacting with the promoters of glycolytic genes, ZFP64 stimulates the transcriptions and subsequently drives glycolysis-mediated stem cell-like properties of breast cancer cells (64). Meanwhile, ZFP64 has been testified to repress immunosuppressive microenvironments in hepatocellular and gastric carcinomas (65, 66). However, ZFP64 has not been systemically studied in BLCA. Our bioinformatic calculations not only further validated the pro-tumoral roles of RBP7 and SREBF1 in BLCA, but also newly linked ZFP64 enrichment to unfavorable prognosis in BLCA patients. MR analysis can minimize confounding and reduce reverse causality to the greatest possible extent, providing genetic-level supportive evidence for these associations.

With the construction of the prognostic signature, BLCA patients were divided into LRG and HRG in this research, which exhibited 12 signaling pathways differentially enriched. Notably, mTORC1 and EMT pathways were significantly activated in the HRG. Containing scaffold protein regulatory-associated protein (RAPTOR) that triggers phosphorylation of downstream proteins, mTORC1 dominates cellular anabolism and promotes cell metabolism, growth, and proliferation of BLCA cells with various target effectors regulated (67). Fundamentally defined by the dissolution of characteristic epithelial features, including cell-cell adhesion and apical-basal polarity, EMT is a key process in cancer metastasis, facilitating malignant cells to adopt a mesenchymal phenotype including strengthened inherent motility and invasiveness, and to acquire the capacity to dissociate from the basement membrane and infiltrate adjacent tissues (68). Besides, a higher enrichment of G2M checkpoint in the HRG implied a hyperactive state of cell proliferation (69). On the other hand, a higher activity of interferon-alpha (IFNα) response was observed in the LRG, which has been reported to reflect a more inflamed microenvironment in MIBC and to predict better responses to immunotherapies (70). Therefore, differences in pathway enrichment between the two risk groups suggest that this risk signature may reflect certain prognosis-related biological differences and provide clues for subsequent mechanistic studies and individualized treatment hypotheses.

The management of advanced BLCA, historically reliant on radical cystectomy and chemotherapy, remains a significant clinical challenge due to its incurable nature and poor long-term outcomes (71). The advent of immune checkpoint blockade (ICB) has introduced a novel treatment paradigm and become the most widely applied immunotherapeutic approach (72, 73). However, the efficacy of ICB is not universal, with objective responses observed only in a minority of patients (73, 74), underscoring the necessity of more comprehensive profiling of immune microenvironments to offer a window into the essential characteristics of pathological defense mechanisms for this neoplasm. Between the two groups defined by the signature in our study, infiltrations of 16 immune cells were detected with differences, among which regulatory T cells (Tregs), mast cells and myeloid-derived suppressor cells (MDSCs) notably presented higher infiltration levels and relatively larger fold changes in the HRG. Maintaining the balance between autoimmunity and immune suppression in humans, Tregs accumulate in tumors to inhibit anti-tumor immunity by weakening T cell activation and act as one of the most important targets for cancer immunotherapy (75). Similarly, MDSCs are recruited to the tumor microenvironment (TME), where they not only potently suppress the anti-tumor activity of T cells and natural killer (NK) cells but also simultaneously performs multiple tumor-promoting effects, resulting in resistance to cancer therapies (76). Mast cells have been reported to have pro-tumorigenic roles with diverse mechanisms in BLCA and are considered pivotal targets for enhancing intravesical Bacillus Calmette–Guérin (BCG) instillation therapy for non-muscle invasive BLCA (77). However, multiple T-cell subsets, like activated CD4+ T cells, central memory CD8+ T cells and effector memory CD4+ T cells that have been known with potent antitumor immunological activities (78–80) also exhibited higher enrichment in the HRG, but with relatively smaller foldchanges compared to the Tregs hyper-infiltration. Destruction of the balance between Tregs and other T cells has been considered a driving force for TME disturbance and immune escape (81). Therefore, the risk score may have a certain association with immunosuppressive features in the BLCA TME. Meanwhile, we observed that 15 immune checkpoint genes were overexpressed in the HRG, whereas only two showed lower expression, suggesting that the HRG may have increased immune checkpoint expression and an immunosuppressive tendency. The elevated expression of the checkpoint molecule TNFRSF18 may partially explain the hyperactive state of Tregs in HRG samples to an extent (81).

Current evidence has linked advanced BLCA patients’ heterogeneous immunotherapeutic responses to tumor mutational burden (TMB) (82). Results of somatic mutation analyzes showed that a higher TP53 mutation frequency was observed in the HRG, which could be responsible for the strengthened malignant phenotype, immune suppression and worse outcome (83, 84). Besides, the frequency of KMT2D mutation in the HRG was 33%, while that in the LRG was 23%. As a potent tumor suppressor, KMT2D inhibits BLCA cell proliferation and metastasis via stimulating H3K4me1 activity (85). Frequently observed in most malignancies, KMT2D mutation triggers BLCA muscle invasion and predicts unfavorable prognosis (86), but it may sensitize tumors to ICB by augmenting tumor immunogenicity (87). Interestingly, samples in the LRG instead of HRG showed FGFR3 mutations, which are potent tumorigenic alterations that facilitate EMT and interfere with TMEs. Thus, HRG patients may not benefit from the application of Erdafitinib, the selective FGFR inhibitor (88). Moreover, we speculated that BLCA patients in different risk groups might exhibit different responses to immunotherapy. According to the following results, 18 immunotherapy-related predictive pathways showed significantly different activity scores between the HRG and LRG. Furthermore, patients in the HRG were associated with higher stromal and ESTIMATE scores, indicating more active stromal cells participating in tumor proliferation, invasion and metastasis in their TMEs as well as an increasing probability of tumor immune escape for this risk group. A certain positive correlation between the risk score and the TIDE prediction score suggests that the poorer prognosis of HRG patients may have a potential association with unfavorable immunotherapy-related predictive features. Notably, the two risk groups show differences in predicted drug sensitivity, which provides clues for further exploration of individualized BLCA treatment strategies based on risk stratification.

In subsequent analyzes, scRNA-seq analyzes were performed to further characterize individual cells and to delineate the cellular mechanisms underlying BLCA tumorigenesis or progression. Eight distinct cell types were identified, comprehensively delineating the cellular landscape and intratumoral heterogeneity of BLCA. T cells, exhibiting different proportions and expression levels of all three prognostic DRRGs between BLCA and normal samples, were identified as potential key cells related to prognostic DRRG expression. We also found that intratumoral T cells were functionally enriched in electrical transmission across gap junctions. Essential to the physiological activities of multicellular animals, gap junctions are formed by connexins, facilitating intercellular communication (89). In the past few years, the alteration of gap junctional intercellular communication or connexin expression has been identified to affect BLCA carcinogenesis, but with context-dependent roles (90, 91). Furthermore, interactions were observed between T cells and the other six cell subtypes, except mast cells. Cancer-associated fibroblasts (CAFs) have been reported to stimulate tumorigenesis and cancer progression via both amplifying Tregs’ immunosuppressive activities and inhibiting effector T-cell functions (92). A recent study has sketched the structured epithelial–T cell communication and related signaling pathways in colorectal cancer, which remodels TMEs and regulates tumor advancement (93). Therefore, T cells are postulated to play a pivotal role in regulating the biological processes of BLCA based on the extensive intercellular crosstalk observed, although the specific mechanisms warrant further investigation. In addition, pseudo-time analysis portrayed the developmental trajectory of intratumoral T cells, providing insights into the dynamic cellular processes underlying BLCA tumorigenesis and progression.

Notably, despite being a risk factor for BLCA patients, RBP7 was observed at reduced abundance in the TCGA-BLCA samples at the mRNA level. Meanwhile, the scRNA-seq analysis detected fewer cells expressing RBP7 compared with the other two DRRGs. We consider that this finding does not necessarily negate the prognostic value of RBP7, because differential expression analysis between tumor and normal tissues and survival-related analysis within tumor patients reflect biological questions at different levels. RBP7 may reflect specific tumor subgroups, differences in cellular composition, tumor microenvironment-related features, or post-translational regulation and downstream pathway feedback, rather than simply showing overall high expression in tumor tissues (94, 95). Therefore, RBP7 may still serve as a prognosis-related variable in the risk model, but its specific biological role and relationship with BLCA progression require further investigation. Therefore, SREBF1 and ZFP64 were prioritized for the subsequent functional experiments in this study. Via our experiments, verified as overexpressed in BLCA tissues at both transcriptome and protein levels, these two DRRGs positively regulated the *in vitro* and *in vivo* malignant phenotypes of BLCA cells, including proliferation, migration, invasion and metastasis. More importantly, the promotion of EMT by SREBF1 and ZFP64 was further supported by western blot results. Recently, SREBF1, the long-known transcription factor, has been reported to accelerate BLCA growth via strengthening fatty acid synthesis (62, 96), but its impact on metastasis-related behaviors remains unknown. On the other hand, a study claimed that SREBF1 protein inhibitor can also restrict cellular glycolytic activity in hepatocellular carcinoma (97). Unstudied in BLCA so far, ZFP64 has emerged as a potent regulatory element in multiple malignancies with different mechanisms involved, including ferroptosis inhibition, glycolysis stimulation, PD-1 and CTLA-4 transcriptional activation as well as TME remodeling (64, 66, 98, 99). Hence, it is worthy of more comprehensive investigation into the specific mechanisms of how these two DRRGs regulate metabolism and TMEs, especially the immune microenvironments in BLCA. Nevertheless, our study not only offered more insights into SREBF1’s impact on BLCA biological phenotypes, but also uncovered the role of ZFP64 in this malignancy for the first time, providing potential therapeutic targets.

Several limitations of this study should be acknowledged. First, the DRRGs were derived from a computationally inferred gene set in a previous pan-cancer analysis and have not been directly validated under dietary restriction conditions in BLCA. Therefore, their DR-relatedness should still be understood as a candidate gene classification and transcriptional-feature association, rather than evidence that they participate in dietary restriction mechanisms in BLCA. Future studies should further validate these findings in DR intervention models, such as calorie restriction or fasting-mimicking diets, by assessing tumor growth, candidate gene expression, and AMPK/mTOR pathway activity. Second, this study was mainly based on retrospective public data. Although the risk model showed certain survival stratification ability in TCGA-BLCA and GSE13507, the AUC values were generally 0.60–0.66, suggesting limited discriminatory ability; therefore, the model is currently more suitable as an exploratory risk-stratification tool. Future studies still need to validate its stability, generalizability, and incremental value over baseline clinical models in a second independent RNA-seq cohort, multicenter cohorts, and prospective cohorts. Third, immune infiltration, TIDE/ESTIMATE, mutation, and drug sensitivity analyzes are all computational inferences and cannot directly represent real immunotherapy or drug treatment outcomes. In addition, because immunotherapy cohorts such as IMvigor210 mainly reflect treatment-specific outcomes in patients with advanced or metastatic urothelial carcinoma receiving immune checkpoint inhibitors, their outcome definitions are not fully consistent with those of TCGA-BLCA and GSE13507. Therefore, this study does not incorporate such cohorts into the current prognostic model validation framework. However, IMvigor210 or similar immunotherapy cohorts can still provide important complementary evidence for the treatment-predictive value of the risk score and may help translate the current TIDE/ESTIMATE-based correlative results into outcome-anchored validation evidence. Future studies can further evaluate the relationship between this risk score and real immunotherapy benefit based on a prespecified immunotherapy validation strategy. Fourth, the animal experiments did not include formal *a priori* sample-size estimation or systematic blinded assessment, and only female BALB/c-nude mice were used; therefore, the *in vivo* results remain preliminary functional validation and should be further confirmed in animal experiments including both sexes, rigorous randomization, and blinded assessment. Fifth, owing to the limited clinical information available in public databases, the multivariable analyzes did not adequately adjust for potential confounders such as M stage, tumor grade, lymphovascular invasion, treatment modality, and smoking status. Finally, the MR effect sizes were small and the GWAS data were mainly from European populations; the T-cell-related findings suggested by scRNA-seq also lack immune functional experimental validation. In addition, some analyzes used raw *p* values as exploratory thresholds, which may increase the risk of false positives. Therefore, the current results should be regarded as exploratory findings and still require further validation using DR intervention experiments, real treatment outcome cohorts, immune coculture systems, and more stringent statistical strategies.

## Conclusions

Overall, our study screened RBP7, SREBF1, and ZFP64 as prognostic DRRGs in BLCA. Subsequently, a prognostic prediction model with certain predictive performance was established as a potential tool for risk stratification of BLCA patients and may provide a reference for individualized management hypotheses. In addition, scRNA-seq analysis suggested that intratumoral T cells are the potential key cell type related to prognostic DRRG expression, depicting the cellular landscape of this malignancy and the dynamic cellular processes involved in carcinogenesis and tumor progression. More importantly, SREBF1 and ZFP64 were validated to be overexpressed in BLCA, and knockdown of these two DRRGs was shown to inhibit the malignant biological behaviors of BLCA cells *in vitro* and *in vivo*, providing clues for subsequent studies.

## Data Availability

The raw data supporting the conclusions of this article will be made available by the authors, without undue reservation.

## Glossary

AUC: area under the curve
BCG: Bacillus Calmette-Guérin
BCA: bicinchoninic acid
BLCA: bladder cancer
CAFs: cancer-associated fibroblasts
CCK-8: Cell Counting Kit-8
CI: confidence interval
CR: calorie restriction
DEGs: differentially expressed genes
DMEM: Dulbecco’s modified Eagle’s medium
DR: dietary restriction
DRRGs: dietary restriction-related genes
EdU: 5-Ethynyl-2′-deoxyuridine
EMT: epithelial-mesenchymal transition
eQTLs: expression quantitative trait loci
FBS: fetal bovine serum
GEO: Gene Expression Omnibus
GO: Gene Ontology
GSEA: Gene Set Enrichment Analysis
GSVA: Gene Set Variation Analysis
GWAS: genome-wide association study
H&E: hematoxylin and eosin
HR: hazard ratio
HRG: high-risk group
HRP: horseradish peroxidase
ICB: immune checkpoint blockade
IC50: half-maximal inhibitory concentration
IVs: instrumental variables
IVW: inverse variance weighted
KEGG: Kyoto Encyclopedia of Genes and Genomes
K-M: Kaplan-Meier
LASSO: least absolute shrinkage and selection operator
LOO: leave-one-out
LRG: low-risk group
MDSCs: myeloid-derived suppressor cells
MIBC: muscle-invasive bladder cancer
MR: Mendelian randomization
NES: normalized enrichment score
NK: natural killer
OR: odds ratio
OS: overall survival
PCA: principal component analysis
PCs: principal components
PH: proportional hazards
PMSF: phenylmethylsulphonyl fluoride
PPI: protein-protein interaction
PVDF: polyvinylidene fluoride
qRT-PCR: quantitative real-time PCR
ROC: receiver operating characteristic
scRNA-seq: single-cell RNA sequencing
shRNA: short-hairpin RNA
SNPs: single nucleotide polymorphisms
ssGSEA: single-sample Gene Set Enrichment Analysis
TCGA: The Cancer Genome Atlas
TIDE: tumor immune dysfunction and exclusion
TMB: tumor mutational burden
TME: tumor microenvironment
Tregs: regulatory T cells
UMAP: uniform manifold approximation and projection
