# Supplementary material for: Identification and experimental validation of prognostic genes associated with dietary restriction-related signatures in bladder cancer
Source: Front Immunol. 2026 Sep 11;17:1891824. doi: 10.3389/fimmu.2026.1891824 (PMC13612542; doi:10.3389/fimmu.2026.1891824)
Supplement: Supplementary file 1 [file Image1.pdf]

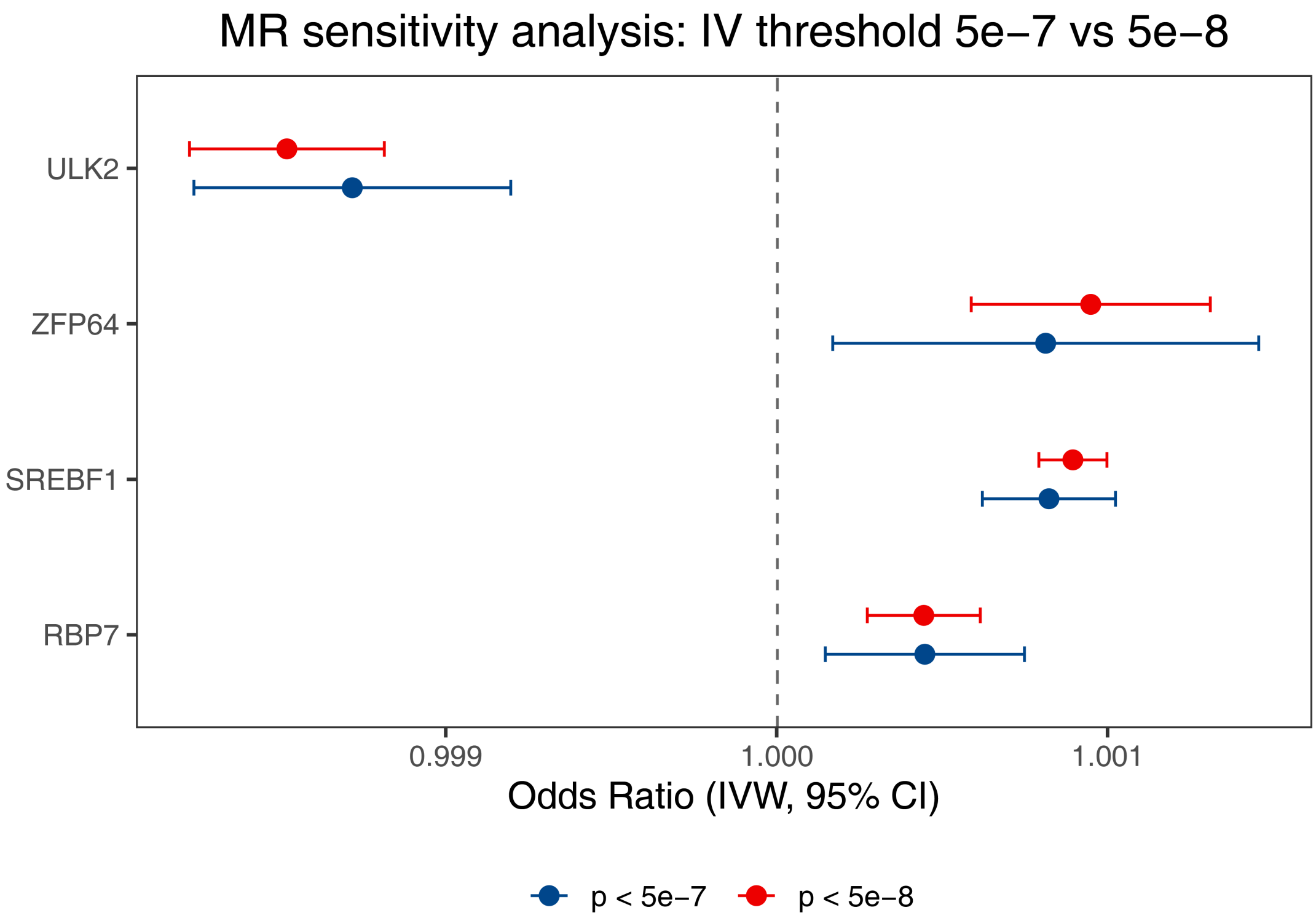

Fig. S1. Forest plot comparing IVW MR effect estimates under instrument-selection thresholds of $p < 5 \times 10^{-7}$ and $p < 5 \times 10^{-8}$.

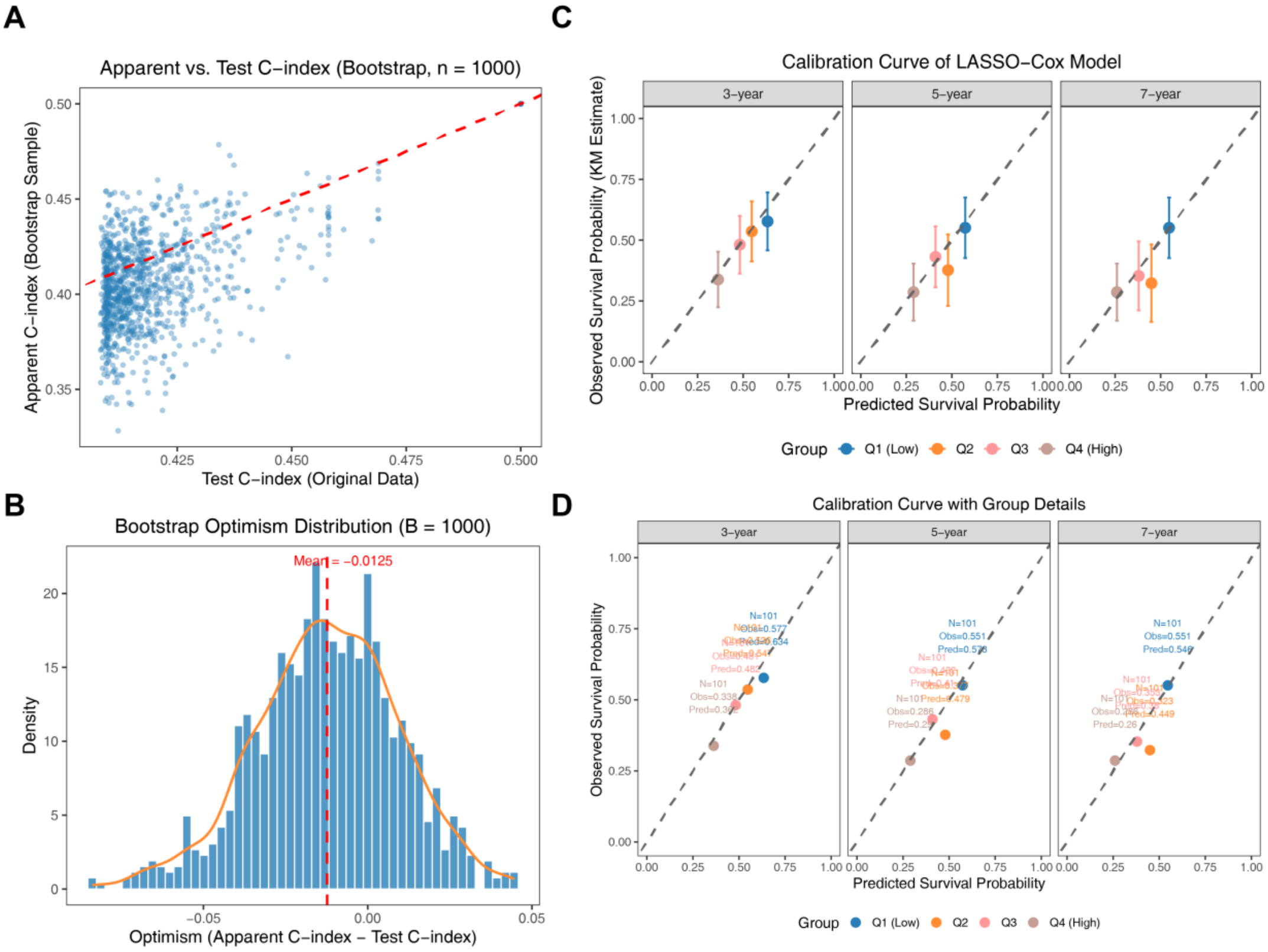

Fig. S2. Internal stability validation of the Lasso model (A-B)bootstrap validation with 1,000 resamples. (C-D) Calibration curves at 3, 5, and 7 years stratified by risk score quartiles.

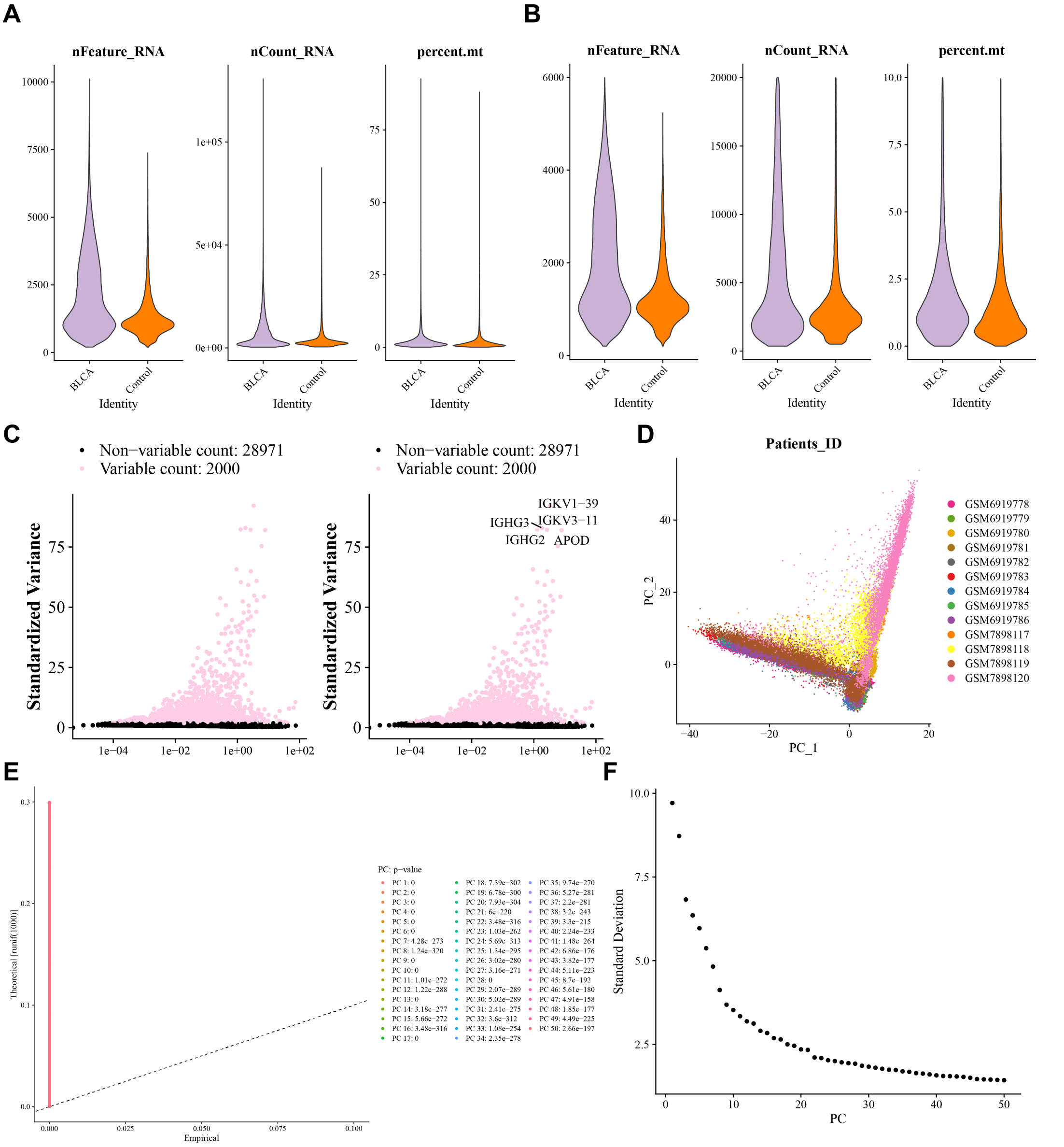

Fig. S3. T cells communicated with a variety of cell types. **A, B** The distributions of the indicated parameters in BLCA and control samples before (A) and after (B) QC. **C** Identification of highly variable genes. **D** The PCA plot. E PCs of BLCA at $p < 0.05$. **F** The ElbowPlot selecting a cutoff of 30 for cell cluster analysis.

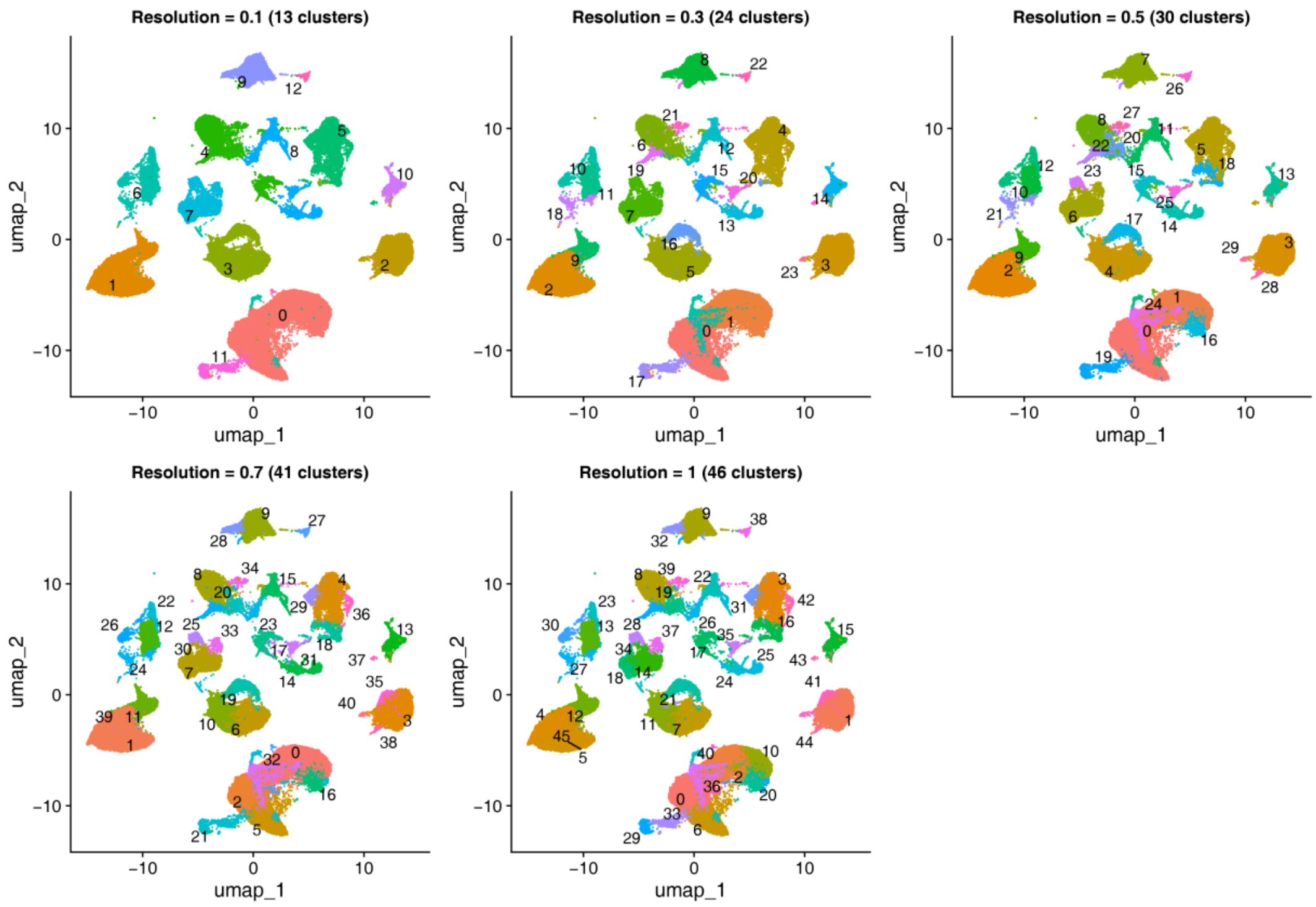

Fig. S4. Robustness assessment of scRNA-seq clustering across different resolution parameters.

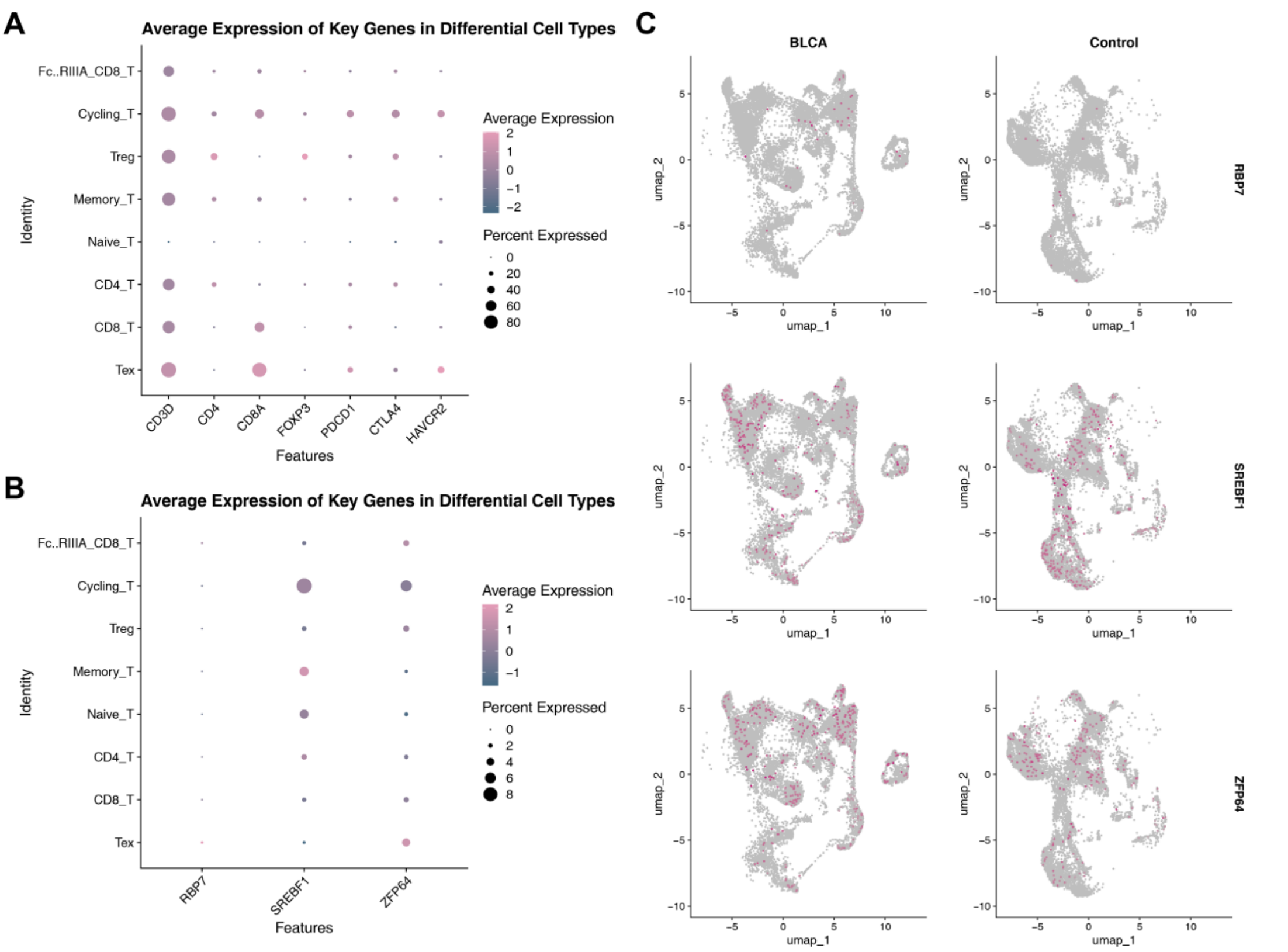

Fig. S5. Expression characteristics of classical functional markers and prognostic DRRGs in different T-cell subclusters.

(A) Dot plots displaying the average expression levels (indicated by color intensity) and the percentages of cells expressing the genes (indicated by dot size) for classical T-cell functional markers (CD3D, CD4, CD8A, FOXP3, PDCD1, CTLA4, and HAVCR2) across distinct T-cell subclusters.

(B-C) Expression distribution of the three prognosis-related DRRGs (RBP7, SREBF1, and ZFP64) across the T-cell subclusters.
