# Supplementary material for: Identification and experimental validation of prognostic genes associated with dietary restriction-related signatures in bladder cancer
Source: Front Immunol. 2026 Sep 11;17:1891824. doi: 10.3389/fimmu.2026.1891824 (PMC13612542; doi:10.3389/fimmu.2026.1891824)
Supplement: Supplementary file 2 [file Table2.docx]

**Table S2. shRNA sequences**

| shRNA | Sense |
| --- | --- |
| sh-SREBF1#1 | ccggGCATTGAGAGTGAAGACCAGTctcgagACTGGTCTTCACTCTCAATGCtttttg |
| sh-SREBF1#2 | ccggGCAAATCTCTGAAGGATCTGGctcgagCCAGATCCTTCAGAGATTTGCtttttg |
| sh-ZFP64#1 | ccggtgCCAATTCAAGACTGCTTATctcgagATAAGCAGTCTTGAATTGGcatttttg |
| sh-ZFP64#2 | ccgggcAATCAAACTTTCATTACCActcgagTGGTAATGAAAGTTTGATTgctttttg |

**Table S3. Sequences of qRT-PCR primers.**

| Primers | Forward sequences | Reverse sequences |
| --- | --- | --- |
| SREBF1 | CGGAACCATCTTGGCAACAGT | CGCTTCTCAATGGCGTTGT |
| ZFP64 | GATGCCTTTGTAGCTCACAAGC | GTCTGGGTCTCCGAGGTGAT |
| β‐actin | CATGTACGTTGCTATCCAGGC | CTCCTTAATGTCACGCACGAT |

**Table S4. Information of primary antibodies.**

| Antibodies | Source | Identifier |
| --- | --- | --- |
| SREBF1 | Proteintech | Cat# 14088-1-AP, RRID: AB_2255217 |
| ZFP64 | Proteintech | Cat# 83825-3-RR, RRID: AB_3671410 |
| N-cadherin | Proteintech | Cat# 22018-1-AP, RRID: AB_2813891 |
| E-cadherin | Proteintech | Cat# 20874-1-AP, RRID: AB_10697811 |
| β‐actin | Proteintech | Cat# 66009-1-Ig, RRID: AB_2687938 |
