# Supplementary material for: Identification and experimental validation of prognostic genes associated with dietary restriction-related signatures in bladder cancer
Source: Front Immunol. 2026 Sep 11;17:1891824. doi: 10.3389/fimmu.2026.1891824 (PMC13612542; doi:10.3389/fimmu.2026.1891824)
Supplement: Supplementary file 3 [file Table8.docx]

**Table S10. Summary of eQTL instrument source and cis/trans composition for candidate genes in the main MR analysis.**

| SYMBOL | analysis | n_IVs | n_cis | n_trans | max_dist_kb | eQTL_source | OpenGWAS_prefix | cis_definition |  |  |
| --- | --- | --- | --- | --- | --- | --- | --- | --- | --- | --- |
| RBP7 | p < 5e-7 | 34 | 23 | 11 | 120556.4 | eQTLGen Consortium (whole blood, n=31,684, European) | eqtl-a (eQTLGen phase 1) | SNP within +/-1 Mb of gene TSS |  |  |
| SREBF1 | p < 5e-7 | 27 | 25 | 2 | 43864.5 | eQTLGen Consortium (whole blood, n=31,684, European) | eqtl-a (eQTLGen phase 1) | SNP within +/-1 Mb of gene TSS |  |  |
| ULK2 | p < 5e-7 | 18 | 16 | 2 | 60488.8 | eQTLGen Consortium (whole blood, n=31,684, European) | eqtl-a (eQTLGen phase 1) | SNP within +/-1 Mb of gene TSS |  |  |
| ZFP64 | p < 5e-7 | 13 | 13 | 0 | 722.7 | eQTLGen Consortium (whole blood, n=31,684, European) | eqtl-a (eQTLGen phase 1) | SNP within +/-1 Mb of gene TSS |  |  |

**Table S11. The result of IVW analysis for the four DRRGs.**

| **gene** | **method** | **Q** | **Q_df** | **Q_pval** |
| --- | --- | --- | --- | --- |
| RBP7 | Inverse variance weighted | 18.01022861 | 33 | 0.98417916 |
| SREBF1 | Inverse variance weighted | 17.10351678 | 26 | 0.90592231 |
| ULK2 | Inverse variance weighted | 14.3043012 | 16 | 0.576055329 |
| ZFP64 | Inverse variance weighted | 2.822270938 | 12 | 0.996674008 |

**Table S12. The result of horizontal multiple validity test for the four DRRGs.**

|  | ENSEMBL | id.exposure | id.outcome | outcome | exposure | egger_intercept | se | pval | SYMBOL |
| --- | --- | --- | --- | --- | --- | --- | --- | --- | --- |
| 1 | eqtl-a-ENSG00000020256 | eqtl-a-ENSG00000020256 | ieu-b-4874 | Bladder cancer \|\| id:ieu-b-4874 | \|\| id:eqtl-a-ENSG00000020256 | -0.000190846 | 0.000158786332942883 | 0.254635890061389 | ZFP64 |
| 2 | eqtl-a-ENSG00000072310 | eqtl-a-ENSG00000072310 | ieu-b-4874 | Bladder cancer \|\| id:ieu-b-4874 | \|\| id:eqtl-a-ENSG00000072310 | -6.37E-05 | 6.51714652393426e-05 | 0.337822353511883 | SREBF1 |
| 3 | eqtl-a-ENSG00000083290 | eqtl-a-ENSG00000083290 | ieu-b-4874 | Bladder cancer \|\| id:ieu-b-4874 | \|\| id:eqtl-a-ENSG00000083290 | -0.000143742 | 8.75981299006969e-05 | 0.121608074175134 | ULK2 |
| 4 | eqtl-a-ENSG00000162444 | eqtl-a-ENSG00000162444 | ieu-b-4874 | Bladder cancer \|\| id:ieu-b-4874 | \|\| id:eqtl-a-ENSG00000162444 | -5.81628E-05 | 5.04936932064446e-05 | 0.25790313209914 | RBP7 |
|  |  |  |  |  |  |  |  |  |  |

**Table S13. The result of horizontal multiple validity test for the four DRRGs.**

| gene | RSSobs | Pvalue |
| --- | --- | --- |
| RBP7 | 18.32761676 | 0.993 |
| ULK2 | 17.8436114 | 0.925 |
| SREBF1 | 17.78493346 | 0.569 |
| ZFP64 | 3.07181512 | 0.996 |

**Table S14. The result of Steiger test for the four DRRGs.**

| gene | id.exposure | id.outcome | exposure | outcome | snp_r2.exposure | snp_r2.outcome | correct_causal_direction | steiger_pval |
| --- | --- | --- | --- | --- | --- | --- | --- | --- |
| SREBF1 | eqtl-a-ENSG00000072310 | ieu-b-4874 | \|\| id:eqtl-a-ENSG00000072310 | Bladder cancer \|\| id:ieu-b-4874 | 0.323203493 | 0.000218196 | TRUE | 0 |
| ZFP64 | eqtl-a-ENSG00000020256 | ieu-b-4874 | \|\| id:eqtl-a-ENSG00000020256 | Bladder cancer \|\| id:ieu-b-4874 | 0.041811418 | 2.37E-05 | TRUE | 2.73E-239 |
| ULK2 | eqtl-a-ENSG00000083290 | ieu-b-4874 | \|\| id:eqtl-a-ENSG00000083290 | Bladder cancer \|\| id:ieu-b-4874 | 0.072533636 | 0.000112597 | TRUE | 0 |
| RBP7 | eqtl-a-ENSG00000162444 | ieu-b-4874 | \|\| id:eqtl-a-ENSG00000162444 | Bladder cancer \|\| id:ieu-b-4874 | 0.207989879 | 7.12E-05 | TRUE | 0 |
|  |  |  |  |  |  |  |  |  |

**Table S17. Results of RBP7, SREBF1 and ZFP64 after BH correction in the differential expression analysis of TCGA-BLCA**

| **Symbol** | **log2FoldChange** | **p value** | **Adjusted p value** | **Change** |
| --- | --- | --- | --- | --- |
| RBP7 | -1.1763 | 0.00237 | 0.00635 | DOWN |
| SREBF1 | 1.1234 | 5.32 × 10⁻⁶ | 2.71 × 10⁻⁵ | UP |
| ZFP64 | 0.6032 | 5.78 × 10⁻⁶ | 2.93 × 10⁻⁵ | UP |

Note: The "Adjusted p value" refers to the p value after correction using the BH method.
